# Systematic benchmark of reduced-lead configurations for 12-lead ECG reconstruction: multi-model evaluation across all possible subsets

**DOI:** 10.3389/fcvm.2026.1856211

**Published:** 2026-06-29

**Authors:** Xinyu Zhang, Hailing Cai, Huilong Duan, Xudong Lu

**Affiliations:** College of Biomedical Engineering and Instrument Science, Zhejiang University, Hangzhou, Zhejiang, China

**Keywords:** 12-lead reconstruction, cardiovascular diagnosis, community care ECG monitoring, composite scoring, electrode efficiency, exhaustive benchmark, lead selection, reduced-lead ECG

## Abstract

Portable reduced-lead ECG devices offer a practical path to pre-hospital emergency care and community cardiovascular screening, yet a foundational question has remained unresolved: which subset of the standard 12 leads should such a device actually record? Prior work evaluates a small number of fixed configurations selected by convention, without systematic comparison across the full combinatorial space or adequate accounting for the true cost of electrode attachment. We present the first exhaustive benchmark evaluating all 4,094 C(12, N) lead subsets for *N* = 1–11 under four reconstruction paradigms—linear regression, ridge regression, a lightweight 1-D convolutional network, and a Transformer encoder–decoder—on the PTB-XL dataset. Performance is assessed along three orthogonal axes: reconstruction fidelity (PCC, RMSE, SNR on withheld leads), downstream diagnostic accuracy (macro-F1 across five cardiac superclasses via a classifier trained on real 12-lead signals), and acquisition efficiency operationalised as electrode contact burden. A Composite Lead Score with five-point α-sensitivity analysis identifies *N* = 4 as the consensus efficiency–accuracy knee (mean macro-F1 = 0.631, 93.5% of the 12-lead upper bound). External zero-shot validation on CPSC2018 (6,877 records) and Chapman-Shaoxing (45,152 records) confirms that all three deployment-recommended configurations retain ≥ 83% of within-PTB-XL three-class F1 on both cohorts (Chapman 92–99%; CPSC 83–91%), and the *N* = 3 knee replicates across all four architectures on Chapman, supporting interpretation of the knee as a property of the lead system's information geometry rather than of any specific architecture or development cohort. A frozen-classifier sensitivity control (*Δ*F1 =  + 0.0006 at *N* = 4 after fine-tuning, an order of magnitude smaller than the cross-N marginal gain) confirms the benchmark's robustness to downstream-model assumptions. Evidence-based configurations are derived for three deployment scenarios: V6 for pre-hospital triage, I + II + AVR + AVF for community screening, and a 7-lead set at 5 contacts for home monitoring; we present these as population-level defaults whose within-tier specific lead choice may benefit from cohort-specific re-derivation. All code and benchmark results are publicly released.

## 1 Introduction

The standard 12-lead ECG remains the cornerstone of cardiovascular diagnosis, offering spatial sampling of cardiac electrical activity from twelve distinct directions and enabling reliable detection of myocardial infarction, bundle-branch block, ventricular hypertrophy, and arrhythmia. However, acquiring a full 12-lead ECG requires specialised hardware, trained personnel, and a controlled clinical environment—conditions that are difficult to satisfy in pre-hospital emergency care, rural community clinics, community-care ECG monitoring programmes, and long-term wearable monitoring. Reduced-lead portable devices offer a practical alternative: they are lightweight, inexpensive, and suitable for continuous acquisition (1, 2). Their diagnostic value is nonetheless limited by the spatial information lost when only a fraction of the standard lead set is recorded.

**Figure 1:**
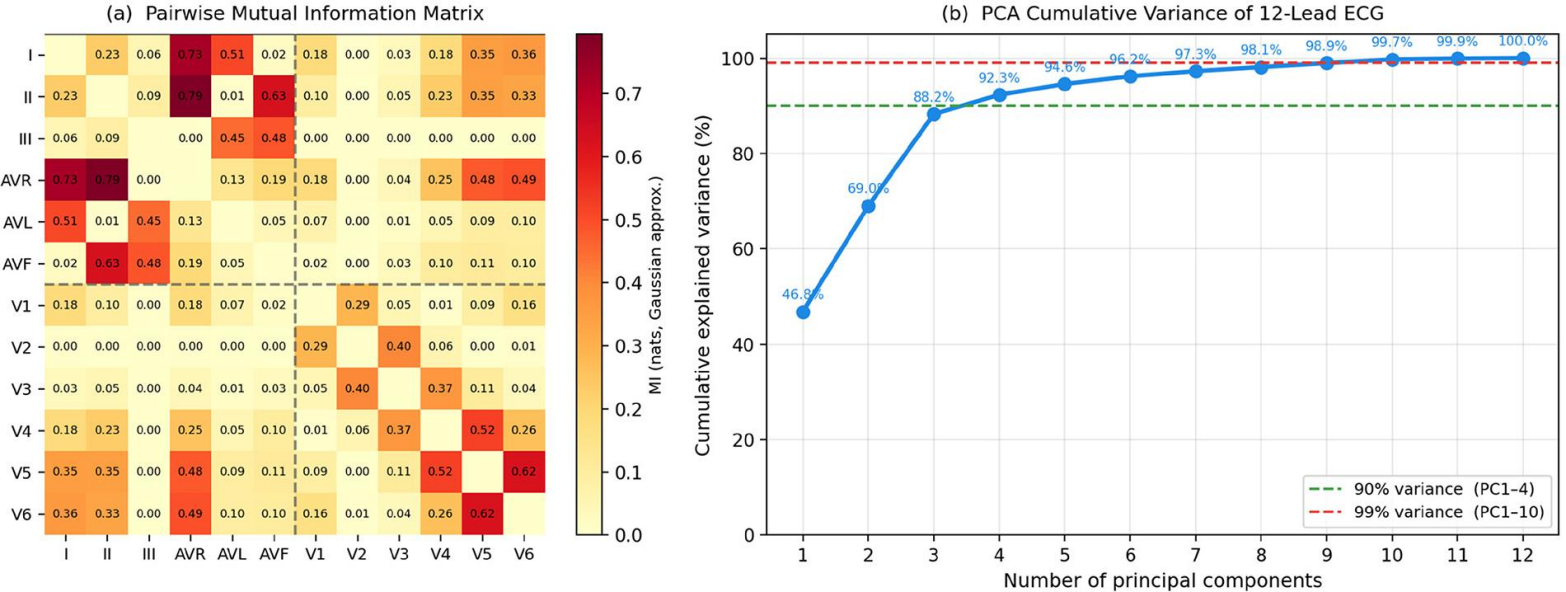
Lead information structure (computed on 3,000 randomly sampled PTB-XL training recordings). **(a)** 12 × 12 pairwise mutual information heatmap [Gaussian approximation: MI ≈ −0.5 log(1−r²)]. Dashed lines separate limb leads (I–AVF, rows/columns 1–6) from precordial leads (V1–V6, rows/columns 7–12). **(b)** PCA cumulative explained variance curve. Green dashed line at 90% (reached at PC 4); red dashed line at 99% (reached at PC 10).

Reconstructing the missing leads from the recorded subset—a task we term ECG information recovery—directly bridges this capability gap. A long line of work has tackled this problem, progressing from hand-crafted linear transformations to data-driven deep learning models based on convolutional networks and Transformers. Despite this progress, a foundational question has been largely overlooked: given that the reconstruction target is always the full 12-lead ECG, which input leads should a portable device actually record? Settling this question on a principled, evidence-based basis is a prerequisite for moving reduced-lead device design beyond convention-driven configurations to designs that quantitatively trade reconstruction fidelity, diagnostic accuracy, and acquisition burden against one another. Virtually all prior studies fix the input configuration *a priori*—typically leads I + II + V3, or the six limb leads, chosen by convention—without comparing alternatives across the full combinatorial space of *N* = 1 to 11 input leads.

A further overlooked dimension is the distinction between lead count and electrode contact burden. In the standard 12-lead system, the six limb leads (I, II, III, AVR, AVL, AVF) are all derived from the same four limb electrodes (RA, LA, LL, RL), while each precordial lead (V1–V6) requires an independent chest electrode placement. Consequently, incrementing lead count by selecting an additional limb lead imposes zero marginal electrode burden if limb electrodes are already attached, whereas adding a precordial lead always incurs an additional contact. Prior efficiency analyses based solely on lead count N therefore systematically misrepresent the true operational cost of different configurations.

These gaps have direct clinical consequences. Single-lead wearables such as the Apple Watch and Kardia Band reliably detect atrial fibrillation (1, 2), but a study of 1,322 hospitalised patients found that the single-lead Kardia Band achieved only 26% sensitivity for non-AF supraventricular tachycardia, with substantially degraded performance on ST-segment and left-ventricular hypertrophy assessment (3). STEMI diagnosis cannot be established from a single lead because clinical guidelines require ST-elevation in two contiguous leads (4); a scoping review confirms that single-lead ECG is fundamentally unsuitable as a STEMI screening tool (5). Expanding to six limb leads (e.g., KardiaMobile 6L) improves arrhythmia discrimination, yet the EVALECG Cardio study (*N* = 1,015) found that the six-limb-lead configuration performed poorly for LVH assessment and ischaemic change detection relative to the 12-lead standard (6)—a predictable consequence of omitting the precordial leads that sample the transverse cardiac plane. Despite this evidence, no systematic study has quantified the diagnostic return of incrementally adding each lead or electrode contact, leaving device designers without an evidence base for lead selection decisions.

**This paper addresses these gaps with three contributions:**

1. Exhaustive combinatorial benchmark. We evaluate all 4,094 C(12, N) lead subsets for N ∈ {1,…,11} under four reconstruction models spanning the linear, convolutional, and attention-based spectrum (linear regression, ridge regression, a lightweight CNN, and a Transformer encoder–decoder), providing the first complete map of how reconstruction fidelity and downstream diagnostic accuracy vary across the entire input-lead space.
2. Electrode-aware multi-dimensional evaluation framework. Each configuration is assessed on three independent axes—reconstruction fidelity (on withheld leads only), downstream diagnostic accuracy (via a classifier frozen on real 12-lead signals), and acquisition efficiency—using an electrode-contact cost model E(C) that correctly accounts for the asymmetric burden of limb vs. precordial placements. A Composite Lead Score (CLS) with a five-point sensitivity analysis translates this framework into clinically actionable recommendations across diverse deployment priorities.
3. Evidence-based configuration decision with external validation. Using Pareto-front optimisation and the Kneedle inflection-point algorithm 7, we identify *N* = 4 as the consensus efficiency–accuracy knee (capturing 93.5% of the 12-lead diagnostic upper bound) and provide a comprehensive reference table of optimal configurations for *N* = 1–11 under multiple clinical priority profiles. We further validate these recommendations under zero-shot transfer to two independent external cohorts (CPSC2018 and Chapman-Shaoxing), demonstrating ≥ 83% F1 retention on shared classes and replication of the *N* = 3 knee across all four architectures.

## 2 Related work

### 2.1 ECG lead reconstruction methods

Early reconstruction methods derived linear mixing matrices from physiological first principles. The Dower transformation and the EASI system express each of the 12 standard leads as a weighted sum of a small set of orthogonal measurements, exploiting the quasi-dipolar nature of the cardiac electrical source. These methods are computationally trivial but impose a single fixed mapping regardless of individual anatomy, leading to systematic errors in patients with unusual cardiac geometry or body habitus.

Deep learning substantially improved reconstruction fidelity. Convolutional encoder–decoder architectures (U-Net variants) capture local morphological patterns in each lead. Generative adversarial networks impose a perceptual quality constraint that reduces high-frequency artefacts. Transformer-based models exploit long-range temporal dependencies and cross-lead attention, achieving state-of-the-art results on PTB-XL. Despite this progress, all of the above methods treat the input lead set as fixed. The reconstruction model is trained and evaluated on a predetermined configuration, without asking whether a different input set might yield better results for the same electrode burden.

More recent work has broadened the architecture palette for ECG reconstruction. Grande-Fidalgo et al. (8) demonstrated a neural-network-based framework for ECG lead reconstruction in ambulatory settings, showing that data-driven models consistently outperform linear transformation baselines on withheld leads. Garg et al. (9) employed an attention U-Net architecture to reconstruct all 12 leads from a single input lead, capturing both morphological detail and long-range temporal context. Beco et al. (10) demonstrated reconstruction of all 12 leads from a single blindly-segmented input using a U-Net framework, establishing a lower bound on input requirements for waveform recovery. Strodthoff et al. (11) provided systematic benchmarks of deep learning architectures for ECG analysis on PTB-XL, showing that model performance degrades non-uniformly across morphological patterns—underscoring the need for reliability-aware deployment in clinical pipelines. Graph convolutional network approaches (12) exploit the inter-lead spatial structure of the ECG as an inductive bias, improving arrhythmia diagnosis on within-group lead sets but offering limited benefit on cross-group (limb↔precordial) transfers where adjacency assumptions break down. Critically, none of the above studies asks which input configuration to use: the lead set is treated as a fixed experimental condition rather than an optimisation variable.

A related but distinct body of work considers reconstruction as a missing-data imputation problem. Methods based on variational autoencoders and diffusion models (13) can synthesise plausible waveform morphology for entirely absent leads by sampling from a learned prior, but these approaches conflate signal synthesis with signal recovery and may introduce hallucinated diagnostic features absent from the actual patient recording. Our benchmark deliberately excludes synthesis-based methods: fidelity is evaluated only on withheld leads, and all models must reconstruct—not generate—missing leads from recorded evidence.

### 2.2 Lead selection and reduction

A separate literature strand considers optimal lead selection for classification rather than reconstruction. Kligfield et al. (14) formalised the information-theoretic argument that the 12 standard leads span a lower-dimensional signal subspace, suggesting significant redundancy. Subsequent studies have used mutual information, principal component analysis, and greedy search to identify compact lead subsets that preserve diagnostic information for specific disease categories—but these studies target classification directly and do not examine reconstruction quality.

For classification rather than reconstruction, several studies have examined which leads carry the most diagnostic information for specific pathologies. Lai et al. (15) showed that an optimally selected 4-lead subset (II, aVR, V1, V4) increases generalizability of deep learning models for ECG abnormality classification, confirming that lead informativeness varies substantially across pathology types—consistent with our disease-subgroup findings in Section 4.7. Strodthoff and Strodthoff (16) demonstrated that a 12-lead convolutional classifier degrades gracefully when leads are masked at inference time, implicitly characterising diagnostic robustness to lead loss, but without systematically covering all C(12, N) subsets or linking results to reconstruction quality. Earlier, Edenbrandt and Pahlm (17) showed that a vectorcardiogram synthesised from the standard 12-lead ECG via inverse transformation retains full diagnostic information, establishing that significant inter-lead redundancy exists in the standard lead system, and Hoekema et al. (18) showed via singular-value decomposition that the 12-lead ECG occupies a subspace of effective rank 8–9. Our PCA analysis recovers consistent findings: 4 principal components explain 90% of signal variance, and 10 explain 99%, bounding the effective dimensionality between these complementary measures.

Wearable and portable ECG systems have created renewed practical urgency around lead reduction. Bayoumy et al. (1) reviewed consumer-grade wearable ECG devices and noted that single-lead patch sensors (typically equivalent to lead I or II) dominate the market, while clinically validated devices mostly use 3–6 leads. Kamga et al. (2) reviewed wearable ECG devices in clinical practice and identified the absence of validated diagnostic algorithms for reduced-lead recordings as a primary barrier to wider clinical adoption. The Kneedle inflection-point algorithm used in our analysis was introduced by Satopaa et al. (7) as a parameter-free method for detecting the elbow point of a monotone curve; we apply it to the N–performance curve to identify the efficiency–accuracy knee without imposing a threshold manually.

To our knowledge, no prior work has (a) systematically evaluated reconstruction quality across the full spectrum *N* = 1–11 under multiple model families, (b) measured fidelity exclusively on the withheld leads, (c) linked results to downstream diagnostic performance via a fixed classifier, or (d) incorporated an electrode-contact cost model to quantify acquisition efficiency accurately. This work fills all four gaps.

## 3 Methodology

### 3.1 Lead combination design

For each input size N ∈ {1, 2, …, 11} we enumerate all C(12, N) lead subsets, yielding 4,094 distinct combinations in total (Table 1). This exhaustive design is the defining methodological contribution of the benchmark: rather than selecting representative configurations by expert judgement, we evaluate the entire combinatorial space and let the data determine which subsets are optimal. Throughout, the standard lead order is: I, II, III, AVR, AVL, AVF, V1, V2, V3, V4, V5, V6 (indices 0–11). Reconstruction fidelity is always evaluated exclusively on the withheld 12−N leads; the N input leads contribute zero reconstruction error by definition and are excluded from all fidelity metrics to prevent artificially inflated scores.

**Table 1:** Exhaustive enumeration of all C(12, N) lead combinations for *N* = 1 to 11.

N	C(12, N)	Cumulative	Notes
1	12	12	Single-lead configurations
2	66	78	
3	220	298	Most prior work evaluated here
4	495	793	
5	792	1,585	
6	924	2,509	Peak combinatorial density
7	792	3,301	
8	495	3,796	
9	220	4,016	
10	66	4,082	
11	12	4,094	Near-complete lead sets
Total	4,094	—	

The “Cumulative” column gives the running total of distinct lead subsets evaluated up to and including each N. Combinatorial density peaks at *N* = 6 with 924 distinct configurations. The “Most prior work evaluated here” annotation at *N* = 3 indicates that conventional reduced-lead reconstruction studies focus their evaluation on three-lead inputs; our benchmark substantially extends this prior coverage by exhaustively evaluating every input size from *N* = 1 to *N* = 11.

### 3.2 Reconstruction methods

To ensure that inflection-point findings reflect lead combination structure rather than artefacts of a particular model family, we evaluate four methods spanning the linear–nonlinear and architecture spectrum (Table 2). All models are trained independently for every combination with no shared weights across configurations, strictly controlling the independent variable (lead subset). All results are checkpointed to individual JSON files after each combination, enabling fault-tolerant resumption of interrupted runs.

**Table 2:** Summary of four reconstruction methods.

Method	Type	Parameters	Key design	Est. time/combo	Coverage
LR	Linear ML	—	Per-timestep OLS: Ŷ = XW	∼10 s^a^	all 4,094
Ridge	Linear ML	—	L2-regularised OLS; *λ* = 1.0	∼10 s*	all 4,094
LightCNN	Deep learning	∼50K	3-layer dilated causal conv 1-D enc–dec	∼15 s	all 4,094
Transformer	Deep learning	∼168K	2 enc + 2 dec, d_model=64, 4 heads, sinusoidal PE	∼45 min	32 representative

a LR/Ridge fitting < 1 s per combination plus ∼8–10 s for downstream classifier inference; total wall time ∼11 h each. LightCNN ∼17 h total. The Transformer reconstructor was evaluated on 32 representative combinations (best PCC, best F1, and best CLS per N) at ∼45 min per combination; full 4,094-combination training was computationally prohibitive. Experiments distributed across two GPU workstations (NVIDIA RTX 4060 and RTX 5060).

**Table 3:** PCC and macro-F1 of best combination at representative N values per model.

Model	*N* = 1 PCC/F1	*N* = 3 PCC/F1	*N* = knee PCC/F1	*N* = 6 PCC/F1	*N* = 11 PCC/F1	Knee N
LR	0.533/0.526	0.822/0.652	0.903/0.671 (*N* = 4)	0.912/0.680	0.855/0.677	4
Ridge	0.533/0.526	0.822/0.652	0.903/0.671 (*N* = 4)	0.912/0.680	0.855/0.677	4
LightCNN	0.765/0.533	0.900/0.654	0.900/0.654 (*N* = 3)	0.950/0.677	0.998/0.678	3
Transformer	0.783/0.558	0.901/0.655	0.901/0.655 (*N* = 3)	0.948/0.676	0.996/0.678	3

Fidelity computed on withheld (12−N) leads only. Transformer values are reported on the 32-configuration evaluation set (per-N best across the configurations evaluated, as described in Section 3.2.4). All inter-N *Δ*F1 steps are statistically significant (bootstrap 95% CI entirely above 0; B = 1,000).

#### 3.2.1 Linear regression (LR)

Per-timestep linear mixing: given the *N* × T input matrix X (*N* leads, T = 1,000 time steps at 100 Hz), we solve Ŷ = XW where W ∈ ℝ^[*N* × (12−N)] minimises the least-squares objective over all training samples and time steps simultaneously. Equivalent to a data-driven Dower-style mixing matrix; serves as the unconstrained linear baseline.

#### 3.2.2 Ridge regression (ridge)

Same per-timestep formulation as LR with L2 regularisation (fixed *λ* = 1.0). Ridge is more robust to collinear leads and small N, and represents the regularised linear ceiling.

#### 3.2.3 Lightweight CNN (LightCNN)

A compact 1-D encoder–decoder with three layers of dilated causal convolutions (∼50 K parameters). Input: *N* × 1,000 tensor. Output: (12−N) × 1,000 tensor. Trained with MSE loss, Adam optimiser (lr = 1e-3), batch size 256, early stopping on validation MSE (patience 5). Masking disabled; each combination is trained only on its designated N input leads.

#### 3.2.4 Transformer encoder–decoder

A lightweight Transformer reconstructor was added in revision to verify that the efficiency–accuracy knee identified by the linear and convolutional models is not specific to those architecture families. The architecture follows the original encoder–decoder formulation (19) with sinusoidal positional encoding: 2 encoder layers and 2 decoder layers, model dimension 64, 4 attention heads, feed-forward dimension 128, totalling approximately 168 K parameters. Inputs are *N* × 1,000 lead time-series; outputs are (12−N) × 1,000 reconstructed leads. Training uses MSE loss on withheld leads, Adam (lr = 1 × 10⁻³ with 1,000-step warm-up), batch size 256, and early stopping on validation MSE (patience 5). Because of the substantially higher per-combination training cost (∼45 min on RTX 5060 vs. ∼15 s for LightCNN), full enumeration of the 4,094-combination space was computationally prohibitive within the revision timeframe. Following the per-N consensus design, we instead trained the Transformer on 32 representative configurations spanning *N* = 1 to 11: for each N ∈ {1, …, 10} we selected three configurations—the best-PCC, best-F1, and best-CLS configuration identified by LightCNN's exhaustive results—and added the two best-F1 configurations at *N* = 11. This sampling enables a faithful reconstruction of the per-N performance envelope, and the knee detected on the resulting Transformer N–F1 curve provides a direct test of architecture-agnostic knee location.

### 3.3 Three-dimensional evaluation framework

All four models are evaluated along three independent axes. Critically, reconstruction fidelity (Dimension 1) is computed exclusively on the withheld 12−N leads to prevent the input leads from trivially contributing zero error.

#### 3.3.1 Reconstruction fidelity (dimension 1)

Per-lead values are averaged across time steps, then across withheld leads, then across test samples. Results are reported separately for limb leads (I, II, III, AVR, AVL, AVF) and precordial leads (V1–V6). Three metrics:

- **PCC** (Pearson Correlation Coefficient): linear correspondence between reconstructed and ground-truth waveform.
- **RMSE** (Root Mean Square Error): amplitude-normalised reconstruction error.
- **SNR** (Signal-to-Noise Ratio, dB): 10 log₁₀(signal power/error power).

#### 3.3.2 Downstream diagnostic accuracy (dimension 2)

A ResNet1D multi-label classifier is trained once on real 12-lead ECG signals from PTB-XL training folds and its weights are frozen for all subsequent experiments (upper-bound macro-F1 on real 12-lead test signals = 0.6751). For each combination, the N input leads are concatenated with the (12−N) reconstructed leads in the standard lead order to form a complete 12-lead input, which is fed into the frozen classifier. We report macro-F1 and per-class F1 for NORM, MI, STTC, CD, and HYP.

#### 3.3.3 Acquisition efficiency (dimension 3)

Quantified not merely as lead count N but as electrode contact burden E, reflecting the actual operational cost of attaching a reduced-lead device to a patient. In the standard 12-lead system, the six limb leads are all derived from the same four electrode positions (RA, LA, LL, RL), while each precordial lead requires an independent chest electrode placement. The electrode contact model is detailed in Section 3.4.

### 3.4 Electrode-contact cost model

We define the electrode contact count E(C) for a lead combination C as:

$$E(C)=4\cdot 1[\exists \;limb\;lead\in C]+|\{V_{1},V_{2},V_{3},V_{4},V_{5},V_{6}\}\;\cap C|$$

where the indicator term captures the fixed cost of attaching all four limb electrodes whenever any limb-derived lead is required, and the second term counts individual precordial placements. E ranges from 1 (a single precordial lead with no limb electrodes) to 10 (all 12 leads requiring 4 limb + 6 precordial contacts).

The two terms of E(C) reflect, respectively, the four-electrode physical limb-bundle [the three active limb electrodes RA, LA, LL plus the right-leg reference, from which all six limb leads I, II, III, AVR, AVL, AVF are linearly derived (14)] and the independent chest-electrode placements required for each precordial lead V1–V6. A full derivation of the cost model from Einthoven's law, together with its mathematical properties and clinical interpretation, is given in Section 5.2 [see also Reyna et al.'s discussion of varying-dimension ECG acquisition in (20)].

This model captures two practically important asymmetries:

(a) All six limb leads (I, II, III, AVR, AVL, AVF) are linearly derivable from three active limb potentials; recording additional limb leads after the first incurs zero additional electrode burden. A configuration using all six limb leads requires only 4 electrode contacts—the same as any single-limb-lead configuration.
(b) Each precordial lead demands precise anatomical chest positioning with gel application (∼20 s per contact), vs. quick limb clamping (∼10 s). The electrode count therefore also serves as a proxy for device preparation time.

The normalised electrode cost is:

$$E_{\mathrm{norm}}(C)=\frac{E(C)-1}{9}$$

### 3.5 Composite lead score and sensitivity analysis

To integrate all three evaluation dimensions into a single, clinically actionable recommendation, we define the Composite Lead Score (CLS):

$$\mathrm{CLS}(C,\alpha )=\alpha \cdot \frac{F_{1}(C)}{F_{1,\mathrm{upper}}}+(1-\alpha )\cdot (1-E_{\mathrm{norm}}(C))$$

where F1(C) is the consensus macro-F1 (mean across LR, Ridge, LightCNN), F1_upper = 0.6751 is the 12-lead upper bound, and α ∈ [0, 1] is the accuracy weight that reflects the clinical priority of diagnostic performance vs. acquisition convenience. Interpretation of α: α → 1 means purely diagnostic priority (ICU, emergency triage); α → 0 means purely efficiency priority (long-term wearable); α = 0.5 is a balanced trade-off.

To avoid dependence on a single arbitrary α choice, we conduct a five-point sensitivity analysis over α ∈ {0.20, 0.35, 0.50, 0.65, 0.80}, corresponding to five representative clinical preference profiles. For each α, we identify the globally optimal combination and the per-N optimal combination, and report the degree to which top recommendations shift across the five settings—yielding a stability metric that informs which configurations are robustly preferred. The F1 in CLS is the consensus mean of the three exhaustively-enumerated model families, deliberately dampening model-specific fluctuations so that CLS reflects the structural information content of the lead combination.

### 3.6 Efficiency–accuracy trade-off analysis

For each model, we compute the mean macro-F1 and mean PCC at each N value, averaged over all C(12, N) combinations. Two complementary methods identify the efficiency–accuracy knee:

#### 3.6.1 Kneedle algorithm

applied independently to the N–F1 curve of each model (7). Statistical significance of the detected knee is assessed by bootstrap resampling (B = 1,000) of the test set; 95% confidence intervals for *Δ*F1 at each N step are reported. Cross-model consensus on the knee location is used as the primary criterion for the final recommendation.

#### 3.6.2 Pareto-Front analysis

for each (*N*, macro-F1) pair—using the best combination at each N—we compute the Pareto front in the (efficiency = 1/N, macro-F1) space. Only Pareto-optimal N values and their best configurations are retained as recommendation candidates.

#### 3.6.3 Mutual information Pre-analysis

the 12 × 12 pairwise mutual information (MI) matrix and PCA cumulative variance curve are computed on the training set prior to the main benchmark to quantify the independent information contributed by each lead.

## 4 Experiments and results

### 4.1 Dataset and experimental setup

All experiments use PTB-XL (21), a publicly available 10-second 12-lead ECG dataset comprising 21,799 recordings sampled at 100 Hz, annotated with SCP-ECG statements mapped to five diagnostic superclasses: NORM (43.6%), MI (25.1%), STTC (24.0%), CD (22.5%), and HYP (12.2%) (multi-label; percentages sum to > 100%). We use the official stratified 10-fold split: training on folds 1–9 (19,601 records), evaluation on fold 10 (2,198 records). The fixed downstream classifier (ResNet1D, trained once on real 12-lead training signals) achieves macro-F1 = 0.6751 on the real 12-lead test set, which serves as the diagnostic upper bound throughout.

The MI analysis reveals that the six limb leads span only two independent dimensions (consistent with Einthoven's law), while the precordial leads V1–V6 contribute approximately four additional independent spatial dimensions. The lead information structure is summarized in Figure 1. Accordingly, the PCA cumulative variance curve shows that only 4 principal components explain 90% of total signal variance, and 10 components are needed to reach 99%—confirming that the effective dimensionality of the 12-lead system is much lower than its nominal rank of 12. A well-chosen set of 6 leads captures approximately 97.6% of the achievable diagnostic accuracy, consistent with this PCA-derived information structure.

### 4.2 Overall N–performance relationship

**Figure 2:**
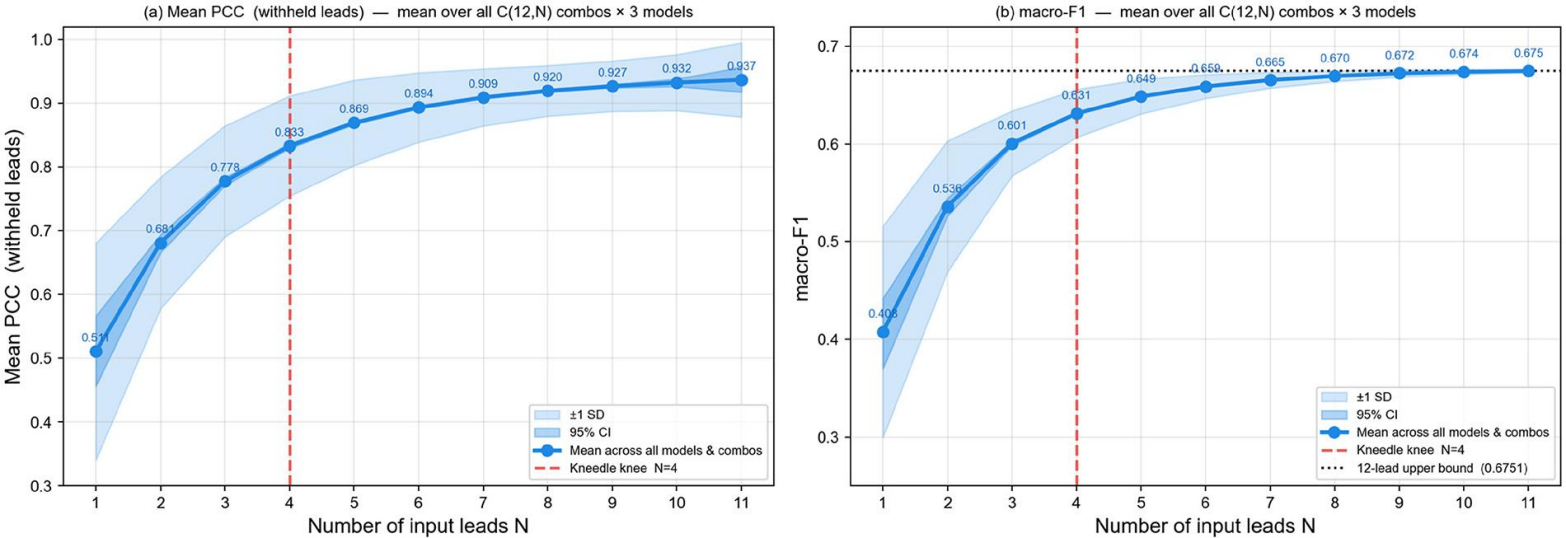
Aggregate N–performance curves [averaged over LR, ridge, LightCNN and all C(12,N) combos]. Left panel: mean PCC (withheld leads only) vs. *N* = 1.11, with ±1 SD shaded band. Right panel: mean macro-F1 vs. N. Upper bound dashed line: F1 = 0.6751. Vertical dashed line at consensus Kneedle knee *N* = 4.

Figure 2 plots PCC and macro-F1 as a function of N averaged across all three models exhaustively enumerated (LR, Ridge, LightCNN); Transformer results on the 32-configuration subset are reported separately in Section 4.3. Each point represents the mean over all C(12, N) combinations with ±1 SD bands.

Across all combinations, both PCC and macro-F1 increase monotonically with N, but marginal gains diminish sharply beyond *N* = 4 (Kneedle-detected consensus knee; LightCNN knee *N* = 3, LR/Ridge knee *N* = 4). The mean macro-F1 rises from 0.408 ± 0.110 at *N* = 1 to 0.631 ± 0.025 at *N* = 4 (93.5% of the 12-lead upper bound), with further gains to 0.659 ± 0.012 at *N* = 6 (97.6%) and 0.675 ± 0.001 at *N* = 11 (99.9%). The largest single-step improvement occurs at *N* = 1 → 2 (*Δ*F1 = 0.128), followed by *N* = 2 → 3 (*Δ*F1 = 0.065); by *N* = 6, each additional lead contributes less than 0.007 in mean F1. The variance across combinations at each N is highest at intermediate values (*N* ≈ 3–6), reflecting the large diversity of available configurations at peak combinatorial density (*N* = 6: 924 combinations).

### 4.3 Per-Model comparison

**Figure 3:**
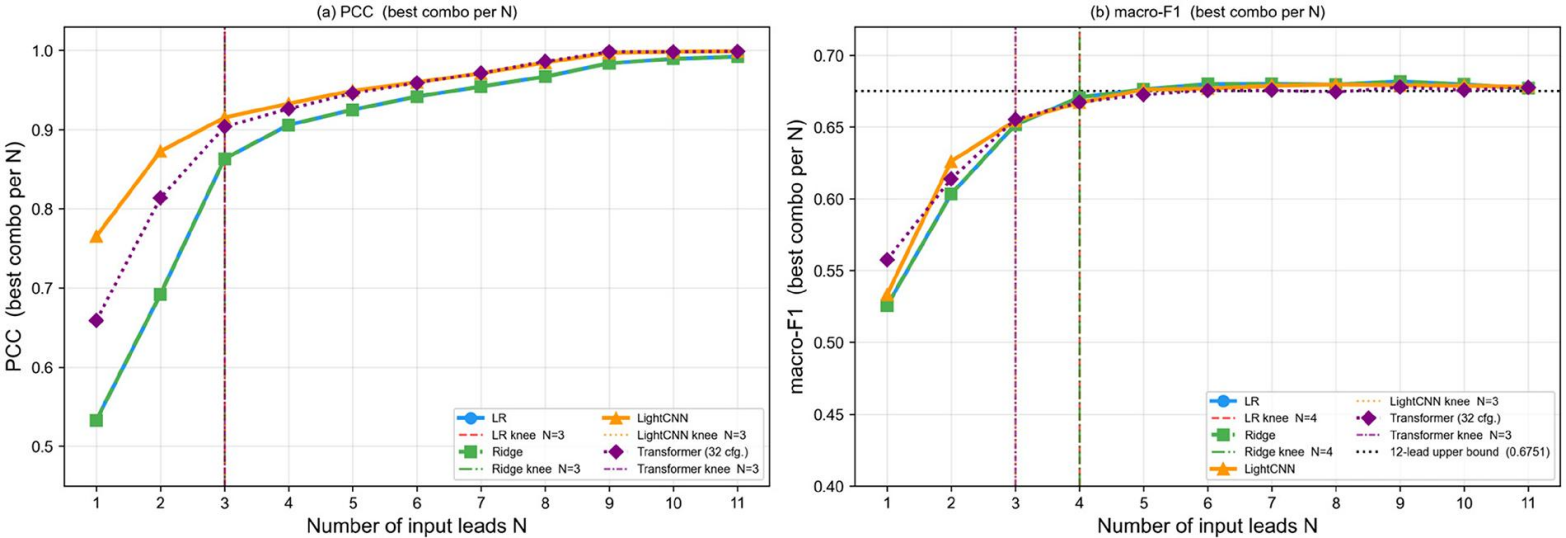
Per-model N–performance curves (best combination per N per model). Left panel: PCC vs. N. Right panel: macro-F1 vs. N. Four lines: LR (blue solid), Ridge (green dashed), LightCNN (orange solid), Transformer (purple dotted). Upper bound dashed line: F1 = 0.6751. Kneedle knee marked per model.

Figure 3 shows N–performance curves disaggregated by model family (LR, Ridge, LightCNN, Transformer), using the best-performing combination at each N for each model.

At *N* = 1, both nonlinear models (LightCNN, Transformer) substantially exceed the linear baselines in PCC (0.765–0.783 vs. 0.533) due to their superior nonlinear inference under extreme input scarcity; however, the F1 gap at *N* = 1 is modest (LightCNN/Transformer 0.533–0.558 vs. LR/Ridge 0.526, *Δ* ≤ 0.032), indicating that diagnostic classification is largely robust to the specific reconstruction architecture even at minimum coverage. At *N* ≥ 6, all four architectures converge to within 0.005 of one another in F1, suggesting that with sufficient input the linear structure of the lead system fully determines reconstruction quality and additional nonlinear capacity provides diminishing benefit.

The key finding from this four-model comparison is the cross-architecture knee consistency: Kneedle detects knee *N* = 4 for LR and Ridge, and knee *N* = 3 for LightCNN and Transformer. The one-lead discrepancy is structurally explainable. The two nonlinear models achieve substantially higher PCC at *N* = 1–3 (∼0.78–0.90 vs. ∼0.53–0.82 for linear models), which compresses the effective gain range of the N–F1 curve and shifts the Kneedle-detected normalised knee one position earlier. Critically, the knee locations of all four architectures fall within the narrow *N* = 3–4 band, and at the recommended *N* = 4 configuration, all four models achieve F1 within 0.005 of their respective *N* = knee values (each model retains ≥ 98% of its per-architecture maximum at *N* = 4). The discrepancy is therefore a marginal feature of normalised inflection detection rather than evidence of model-specific structure; the consensus *N* = 4 is adopted as the benchmark knee throughout. The robustness of this conclusion is further verified by external-cohort replication of the knee analysis in Section 4.8.3, where all four architectures converge to knee *N* = 3 on Chapman-Shaoxing.

All inter-N *Δ*F1 steps are statistically significant under a bootstrap test (B = 1,000 resamples; 95% CI entirely above zero for all *N* = 1 → 2 through *N* = 10 → 11 steps). The three largest gains are: *N* = 1 → 2 [*Δ*F1 = 0.128, 95% CI (0.092, 0.164)], *N* = 2 → 3 [*Δ*F1 = 0.065, 95% CI (0.055, 0.075)], and *N* = 3 → 4 [*Δ*F1 = 0.031, 95% CI (0.028, 0.034)]. Beyond *N* = 6, the per-step gain falls below 0.007 with tight CIs, confirming the saturation regime.

The identity of the specific top-3 lead configurations at each N—ranked separately by macro-F1 and by mean PCC—is visualised in Supplementary Figures S1 and S2 (Supplementary Material). A full numerical lookup table listing the top-3 configurations per N under each optimisation criterion (PCC, macro-F1, and CLS) is provided in Supplementary Tables S1a–c. Representative PCC/F1 values and knee locations for each reconstruction family are summarized in Table 3.

### 4.4 Pareto analysis and lead-count configuration recommendations

**Figure 4:**
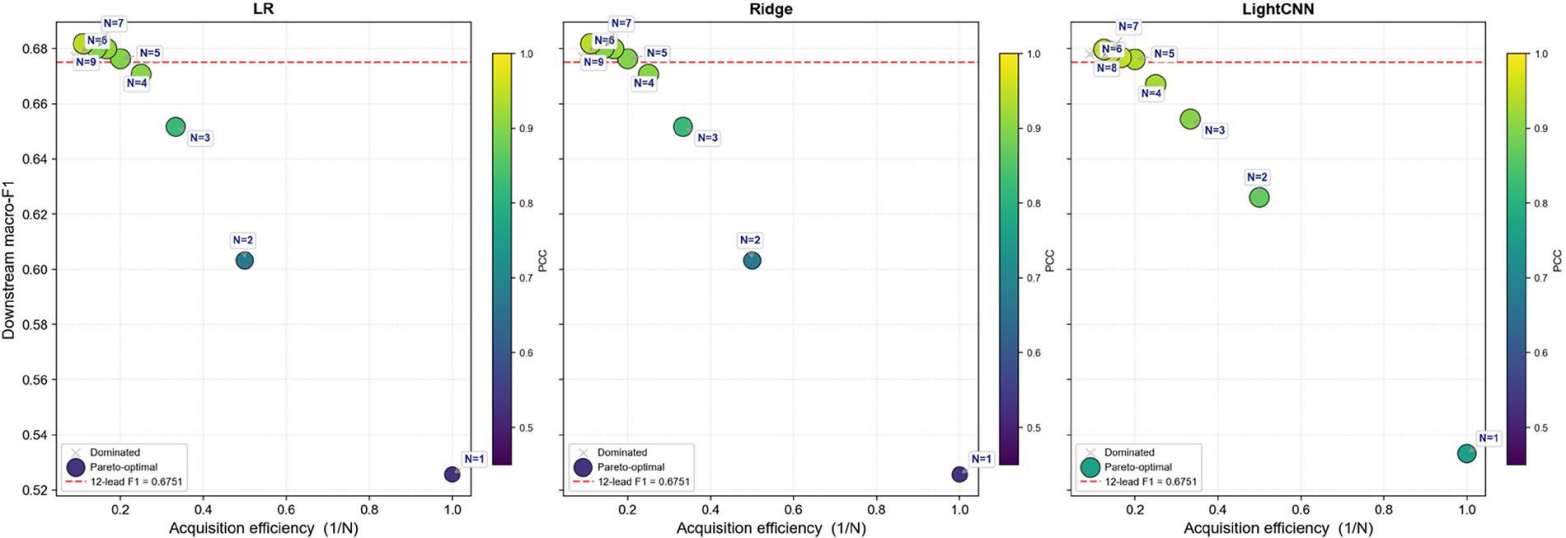
Pareto-front analysis (lead-count efficiency = 1/N). *X*-axis: acquisition efficiency = 1/N. *Y*-axis: macro-F1. Point size = PCC. Three panels (LR, Ridge, LightCNN). Pareto-front points: coloured circles labelled with N and best lead subset. Dominated points: grey crosses. Three clinical scenario recommendations annotated.

Figure 4 shows the Pareto front in the (efficiency = 1/N, macro-F1) space, using the best combination at each N for each model. Point size encodes PCC, allowing simultaneous visualisation of all three evaluation dimensions.

The Pareto front is dominated by configurations with N ∈ {1, 4, 6} across all model families, suggesting that the efficiency–accuracy trade-off structure is largely model-independent. Configurations with *N* > 6 are dominated for all models under the 1/N efficiency proxy. The *N* = 4 configuration (four limb electrodes) sits at the efficiency–accuracy corner of the Pareto front: it achieves 93.5% of maximum F1 at 36% of the maximum lead burden.

### 4.5 Composite lead score and sensitivity analysis

**Figure 5:**
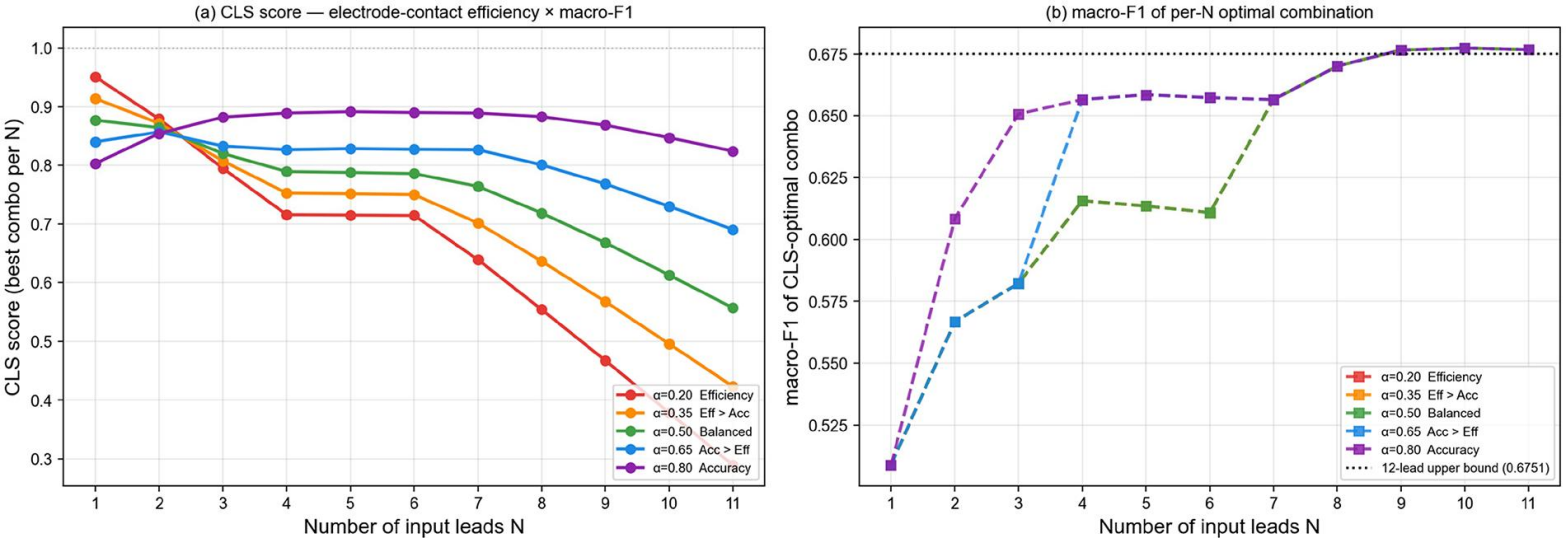
CLS sensitivity analysis (electrode-contact efficiency   ×  macro-F1). **(a)** Composite Lead Score of the per-N optimal combination under five accuracy weights α ∈ {0.20, 0.35, 0.50, 0.65, 0.80}. **(b)** macro-F1 of the corresponding optimal combinations. Dashed horizontal line: 12-lead upper bound F1 = 0.6751. *X*-axis: *N* = 1 to 11.

**Figure 6:**
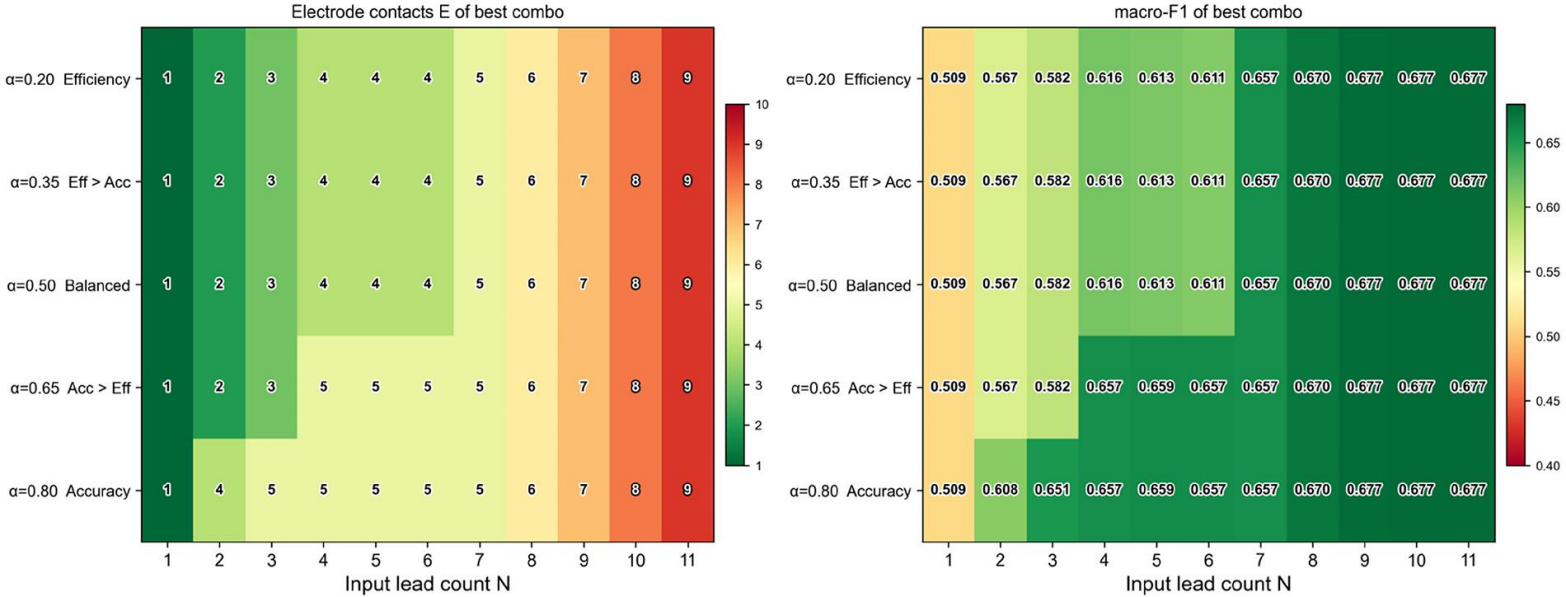
Sensitivity heat maps (weight α × lead count N). Left: electrode contact count E of the per-N, per-*α* optimal combination. Right: macro-F1 of the same combinations. For *N* = 4–6 under low α, the optimal combination is always an all-limb configuration (E = 4), demonstrating the electrode-sharing benefit.

**Figure 7:**
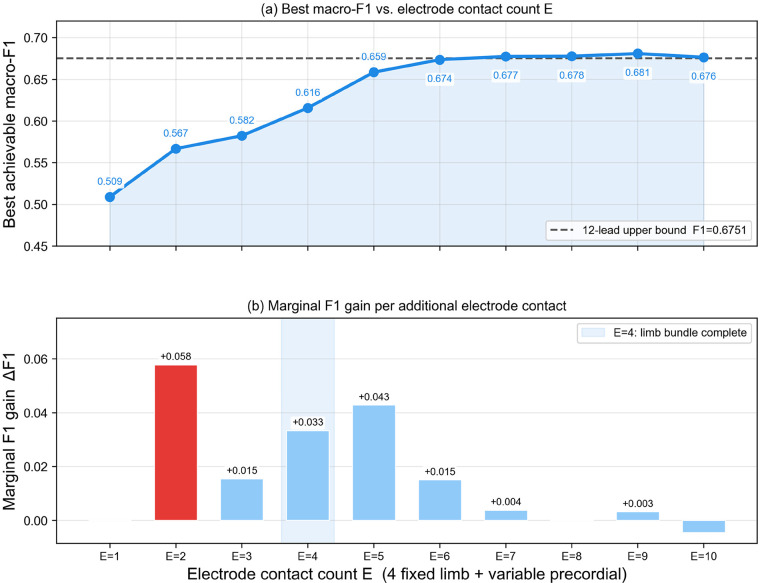
F1 benefit per electrode contact (electrode-based efficiency frontier). **(a)** Best achievable macro-F1 as a function of E (1–10). **(b)** Marginal *Δ*F1 per additional electrode contact. The largest single marginal gain occurs at E = 4 (completing the limb electrode circuit, enabling all six limb leads simultaneously); subsequent precordial contacts contribute progressively smaller increments.

Figure 5 presents the CLS sensitivity analysis results. Panel (a) shows the CLS of the best combination at each N under each of the five α settings. Panel (b) shows the corresponding macro-F1 of those best combinations. Figures 6, 7 show sensitivity heat maps and marginal F1 gain per additional electrode contact.

**Figure 8:**
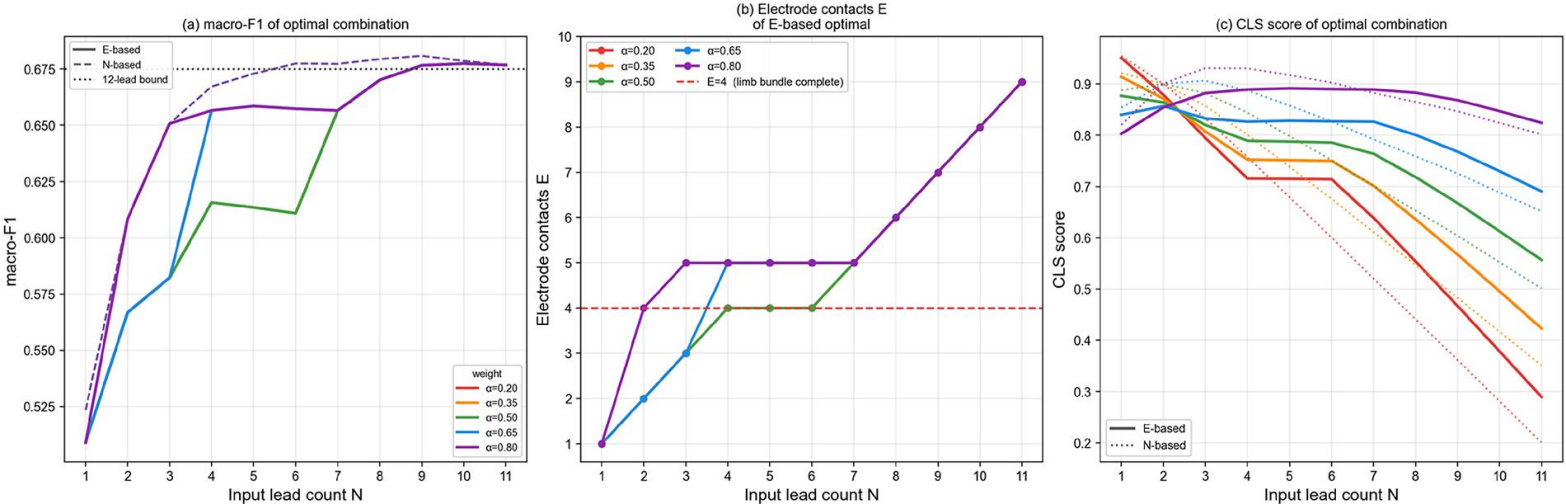
Electrode-contact efficiency (E-based, CLS_E_F1) vs. lead-count efficiency (N-based, CLS_N_F1) comparison at α = 0.50. **(a)** macro-F1 of the per-N optimal combination under each metric (solid = E-based, dashed = N-based). **(b)** Electrode contact count E of the E-based optimal combination at each N. **(c)** CLS scores under both metrics. The E-based model consistently selects lower-F1 but more electrode-efficient configurations at *N* = 4–6.

Table 4, Table 5 present the globally optimal lead combinations under each CLS weight configuration, evaluated with macro-F1 and PCC as the accuracy metric respectively.

**Table 4:** Globally optimal CLS combination per weight (accuracy metric = macro-F1, efficiency metric = electrode contacts E). Consensus F1 and PCC = mean across LR, Ridge, LightCNN.

**Weight config (α)**	**Optimal leads**	**N**	**E**	**macro-F1**	**CLS**
α=0.20 (efficiency)	V6	1	1	0.509	0.951
α=0.35 (moderate eff.)	V6	1	1	0.509	0.914
α=0.50 (balanced)	V6	1	1	0.509	0.877
α=0.65 (moderate acc.)	V2 + V6	2	2	0.567	0.857
α=0.80 (accuracy)	I + III + AVR + AVF + V2	5	5	0.659	0.891

**Table 5:** Globally optimal CLS combination per weight (accuracy metric = mean PCC, efficiency metric = electrode contacts E).

Weight config (α)	Optimal leads	N	E	mean PCC	CLS
α=0.20 (efficiency)	V5	1	1	0.570	0.914
α=0.35 (moderate eff.)	V5	1	1	0.570	0.849
α=0.50 (balanced)	V5	1	1	0.570	0.785
α=0.65 (moderate acc.)	III + AVR + V3	3	5	0.880	0.767
α=0.80 (accuracy)	III + AVR + V2 + V5	4	6	0.914	0.820

**Table 6:** Evidence-based clinical scenario recommendations (CLS_E_F1, α = 0.50).

Scenario	Recommended leads	N	E (contacts)	macro-F1	Rationale
Pre-hospital emergency	V6	1	1	0.509	Minimal setup; single chest patch
Community screening	I + II + AVR + AVF	4	4	0.616	Limb-only; 4 clips, no chest prep
Home monitoring	I + II + III + AVR + AVL + AVF + V1	7	5	0.657	Near-full F1; 1 precordial added

The sensitivity analysis reveals three key findings. First, under efficiency weighting (α ≤ 0.50), the globally optimal combination is a single precordial lead (V6 by F1, V5 by PCC): a single chest electrode requires no limb setup and achieves F1 = 0.509/PCC = 0.570. Second, the transition to accuracy-dominant weighting (α = 0.65–0.80) shifts the optimum to small mixed sets (*N* = 2–5). Third, F1-based and PCC-based CLS rankings diverge for moderate-accuracy profiles: PCC-based scoring favours configurations with higher reconstruction fidelity that do not necessarily maximise downstream F1, highlighting that reconstruction quality and diagnostic accuracy are distinct optimisation targets. A complementary sensitivity analysis on the assumption of uniform precordial electrode cost—under which V1/V2 (slower septal placement) and V3/V4 (faster mid-chest placement) are assigned weights of 1.3 and 0.8 respectively, relative to V5/V6 at 1.0—confirms that the three deployment-recommended configurations remain in the per-N CLS top-3 under both the default uniform weighting and a ± 30% precordial-cost perturbation; complete stability results are given in Supplementary Table S5. The resulting evidence-based clinical scenario recommendations are summarized in Table 6.

### 4.6 Lead-count efficiency vs. electrode-contact efficiency: a comparative analysis

To assess whether the electrode-contact cost model (CLS_E) provides substantively different guidance from the simpler lead-count proxy [CLS_N, efficiency = 1−(*N*−1)/10], we ran a parallel analysis using CLS_N_F1 and compared per-N optimal recommendations at α = 0.50. The E-based and N-based recommendations are compared in Figure 8.

The two metrics yield different recommendations at every *N* value from 1 to 10 (only converging at *N* = 11, where all combinations are identical). The most clinically significant divergence occurs at *N* = 4–6: the N-based model recommends mixed limb-precordial combinations (e.g., I + AVF + V1 + V5 at *N* = 4, macro-F1 = 0.667, E = 5), while the E-based model recommends all-limb combinations with the same or lower N but only 4 electrode contacts (e.g., I + II + AVR + AVF at *N* = 4, macro-F1 = 0.616, E = 4). The E-based recommendation sacrifices approximately 0.05 in F1 to save one electrode contact—a trade-off that may be worthwhile in settings where chest electrode placement is the primary bottleneck (e.g., patient with obesity, skin conditions, or motion artefact sensitivity), but suboptimal in diagnostic settings where F1 is paramount.

This divergence is a direct consequence of the electrode-sharing asymmetry: all-limb configurations at *N* = 5 and *N* = 6 still cost only E = 4 contacts, making them appear highly efficient under the E-based metric even as their F1 plateaus. The N-based metric, by penalising N directly, does not distinguish between a 4-electrode and a 6-electrode configuration, and therefore correctly identifies that adding one chest electrode (E = 5) yields a larger F1 gain than adding a third or fourth redundant limb lead at no electrode cost.

### 4.7 Disease subgroup analysis

**Figure 9:**
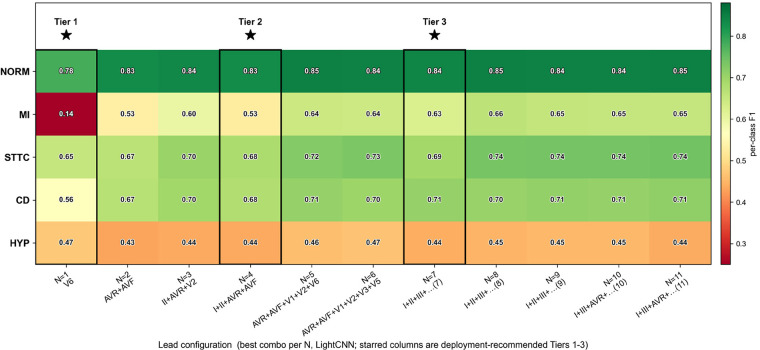
Disease subgroup heatmap (LightCNN). Rows: NORM, MI, STTC, CD, HYP. Columns: top-3 configurations by F1 per N (∼33 columns). Cell colour: per-class F1 (viridis colormap, 0→1). Vertical dividers between N groups. The three deployment-recommended configurations (Tier 1: V6; Tier 2: I + II + AVR + AVF; Tier 3: 7-lead set) are enclosed in bold black bounding boxes with a ★ marker above the column header.

Figure 9 shows per-class F1 heat maps for representative configurations under LightCNN, with columns ordered by N. Table 7 reports per-class F1 for the best LightCNN configuration at each N.

**Table 7:** Per-class F1 at the best LightCNN combination per N.

N	Leads (best combo)	NORM	MI	STTC	CD	HYP	macro-F1
1	II	0.791	0.313	0.601	0.627	0.334	0.533
2	AVR + AVF	0.831	0.533	0.665	0.670	0.430	0.626
3	II + AVR + V2	0.838	0.599	0.703	0.695	0.437	0.654
4	AVR + AVF + V2 + V6	0.845	0.634	0.714	0.687	0.455	0.667
5	AVR + AVF + V1 + V2 + V6	0.846	0.645	0.723	0.706	0.461	0.676
6	AVR + AVF + V1-V3 + V5	0.843	0.643	0.731	0.700	0.466	0.677
7	II + III + AVF + V1-V3 + V6	0.851	0.650	0.732	0.706	0.455	0.679
8	I + II + III + AVL + V1-V3 + V6	0.848	0.663	0.736	0.704	0.448	0.680
11	All leads except II	0.846	0.653	0.742	0.711	0.439	0.678

Fidelity on withheld leads only. Contiguous precordial leads are abbreviated with hyphen notation (e.g., V1-V3 = V1 + V2 + V3).

Several disease-specific patterns are noteworthy. MI: the lowest-performing class throughout (F1 = 0.313 at *N* = 1, rising to 0.663 at *N* = 8), with the steepest gains from *N* = 1 → 2 (+0.220) and *N* = 2 → 3 (+0.066), both driven by introduction of precordial leads (AVF + AVR at *N* = 2; V2 at *N* = 3) that capture anterior and inferior ST-segment changes. This confirms that MI diagnosis is the most lead-hungry task. HYP: similarly low at *N* = 1 (F1 = 0.334) and peaks at *N* = 6 (F1 = 0.466); hypertrophy detection depends on QRS voltage criteria spanning multiple spatial directions. NORM: already reliable at *N* = 1 (F1 = 0.791) and remains near its asymptote beyond *N* = 3, confirming that ruling out gross abnormality requires minimal spatial coverage. CD: moderately robust even at *N* = 1 (F1 = 0.627), suggesting that limb-lead QRS morphology captures the majority of conduction disturbance signatures. STTC: consistent improvement across all N (0.601 → 0.742), reflecting diffuse repolarisation abnormalities that benefit from incremental precordial spatial coverage.

To enable clinicians and device designers to identify the optimal lead configuration for any specific diagnostic priority at deployment-relevant *N* values, Table 8 summarises the per-disease optimal configurations at *N* = 1, 4, and 7—corresponding to the three deployment tiers identified in Section 4.5. The full *N* = 1–11 per-disease table is provided in Supplementary Table S2.

**Table 8:** Per-disease optimal lead configuration at *N* = 1, 4, and 7 (best across LR, ridge, LightCNN).

Disease	*N* = 1 best lead	F1	*N* = 4 best config	F1	*N* = 7 best config	F1
NORM	AVR	0.795	I + AVL + AVF + V2	0.848	I + III + AVR + V1 + V2 + V3 + V6	0.852
MI	III	0.492	I + AVL + AVF + V2	0.679	I + III + AVR + AVF + V2 + V3 + V5	0.680
STTC	V5	0.686	AVL + V3 + V5 + V6	0.753	II + AVR + AVL + V3 + V4 + V5 + V6	0.756
CD	II	0.627	II + AVL + AVF + V1	0.719	II + III + AVR + AVL + V1 + V4 + V6	0.719
HYP	V6	0.472	I + V2 + V5 + V6	0.479	I + II + V1 + V2 + V3 + V5 + V6	0.470

The *N* = 4 configurations differ substantially across disease subclasses, supporting condition-specific lead selection for targeted screening applications.

Two clinically actionable patterns emerge. First, the *N* = 4 optimal configuration is highly disease-specific: the MI-optimal four-lead set (I + AVL + AVF + V2) emphasises septal and inferior coverage to capture acute ST-elevation territories, whereas the STTC-optimal set (AVL + V3 + V5 + V6) prioritises left-precordial repolarisation sampling. A single 4-lead configuration cannot simultaneously optimise all five diagnostic targets, motivating either tiered acquisition strategies or post-deployment classifier-specialisation depending on the screening application. Second, NORM and CD detection reach near-asymptotic F1 already at *N* = 1 (0.795 and 0.627 respectively), confirming that ruling-out workflows can be served by single-lead devices, while MI and HYP—the diagnostically and clinically highest-stake classes—remain the limiting factors that motivate multi-lead configurations.

### 4.8 External validation

To verify that the deployment-recommended configurations identified on PTB-XL transfer beyond the development cohort, we performed zero-shot external validation on two publicly available 12-lead ECG datasets and replicated the efficiency–accuracy knee analysis on the larger external cohort, complemented by exhaustive enumeration of N ∈ {1, …, 6} configurations on that cohort.

#### 4.8.1 Datasets and preprocessing

Two external datasets were selected to span different degrees of distributional shift from PTB-XL:

**CPSC2018** (22), 6,877 records, 12-lead at 500 Hz, drawn from the China Physiological Signal Challenge 2018. Diagnostic labels follow a 9-class scheme dominated by rhythm findings; only NORM, STTC, and CD have direct correspondence to PTB-XL superclasses (MI and HYP are unlabeled in this dataset and cannot be evaluated).

**Chapman-Shaoxing** (23), 45,152 records, 12-lead at 500 Hz, jointly contributed by Chapman University, Shaoxing People's Hospital, and Ningbo First Hospital. Each record carries multi-label SNOMED-CT annotations spanning 94 unique diagnostic codes covering both rhythm and morphology findings.

Both datasets were resampled from 500 Hz to 100 Hz, center-cropped or zero-padded to 1,000 samples (10 s), and z-score normalised per lead using PTB-XL training statistics. Diagnostic codes were mapped to the PTB-XL five-superclass framework using an expert mapping derived from each dataset's bundled diagnostic-code dictionary supplemented by the PhysioNet/CinC 2021 dx_mapping reference (20); full mapping rules are provided in Supplementary Table S3.

For Chapman-Shaoxing, 41.8% of records carry only rhythm-level annotations (sinus rhythm, sinus bradycardia, atrial fibrillation, sinus tachycardia, sinus arrhythmia) without morphological diagnostic codes. Because these records lack assessment for the morphological abnormalities that define MI/STTC/CD/HYP superclasses, the primary analysis is restricted to the 26,264 records (58.2%) carrying at least one mapped morphological label. A sensitivity analysis treating rhythm-only records as NORM (45,150 records, “Chapman sensitivity”) confirms the primary conclusions and is reported alongside.

#### 4.8.2 Zero-shot retention of deployment configurations

For each external cohort, the three deployment-recommended configurations from Section 4.5—V6 (Tier 1, pre-hospital triage; *N* = 1), I + II + AVR + AVF (Tier 2, community screening; *N* = 4), and I + II + III + AVR + AVL + AVF + V1 (Tier 3, home monitoring; *N* = 7)—were evaluated under zero-shot transfer. Reconstruction models trained on PTB-XL were applied to external inputs without any retraining, reconstructed 12-lead signals were passed through the frozen ResNet1D classifier, and macro-F1 was computed on the shared three-class subset (NORM, STTC, CD) to enable apples-to-apples comparison across cohorts despite CPSC's partial label coverage. The reported metric is F1 retention, defined as the ratio of external macro-F1 to PTB-XL macro-F1 computed on the same configuration and same three classes; this within-configuration denominator isolates cohort effects from absolute model performance.

All three recommended configurations retain ≥ 83% of the within-PTB-XL three-class F1 on both external cohorts. Chapman retention exceeds CPSC retention by approximately 10 percentage points across all configurations, consistent with Chapman's closer alignment with PTB-XL in clinical-acquisition setting and multi-label morphological annotation coverage, vs. CPSC's narrower competition-derived labeling. The Tier-1 single-lead V6 configuration retains 91% (CPSC) and 99% (Chapman) of three-class F1, supporting its viability for pre-hospital triage in distributionally varied settings, and in fact exceeds direct 12-lead classification on Chapman—a phenomenon discussed in Section 4.8.5 below.

The Chapman primary-versus-sensitivity comparison (right two columns of Table 9) confirms that the morphology-subset restriction does not bias retention conclusions: absolute retention drops by 5–7 percentage points under inclusive labeling, but the ranking of configurations and the relative spacing among tiers are preserved.

**Table 9:** Zero-shot three-class F1 retention (NORM/STTC/CD) of deployment-recommended configurations on external cohorts.

Configuration	Tier	CPSC2018	Chapman primary	Chapman sensitivity
V6	1 (pre-hospital)	91%	99%	92%
I + II + AVR + AVF	2 (community)	88%	98%	89%
7-lead set	3 (home)	83%	94%	90%

Reported values use LR/Ridge reconstruction (the linear baseline; CNN values differ by <5 pp on Chapman and ∼15 pp lower on CPSC due to overfitting, see Supplementary Table S4).

#### 4.8.3 Replication of the efficiency–accuracy knee on chapman-shaoxing

To verify that the *N* = 3–4 knee identified on PTB-XL is a property of the lead system rather than of the development cohort, we performed two complementary analyses on Chapman-Shaoxing primary subset.

##### 4.8.3.1 Approach 1—Per-N best-PTB-XL-config transfer

For each N ∈ {1, …, 11} and each model, the configuration achieving the highest F1 on PTB-XL was applied to Chapman without retraining, and Kneedle (7) was used to localise the knee on the resulting Chapman N–F1 curve.

##### 4.8.3.2 Approach 2—full chapman enumeration for N ∈ {1, …, 6}

To assess robustness against the specific transfer choice in Approach 1, we exhaustively enumerated all C(12, N) configurations for N ∈ {1, …, 6} on Chapman primary, yielding 2,509 combinations per model for LR and Ridge. Computational cost prohibited full enumeration for LightCNN; we instead evaluated 878 configurations comprising all combinations at *N* = 1 (12) and *N* = 2 (66), and the top-200 PTB-XL-F1 configurations at each of *N* = 3.6.

Both approaches yield the same conclusion (Table 10): on Chapman, the efficiency–accuracy knee is at *N* = 3 for all four reconstruction architectures, within one lead of the PTB-XL knee at *N* = 3–4. For LR/Ridge, Chapman F1 saturates between *N* = 3 (0.701) and *N* = 6 (0.709), a 1.2-percentage-point absolute gain over three additional leads; for LightCNN, F1 plateaus at *N* = 3 (0.714) and remains at 0.714 through *N* = 6.

**Table 10:** Knee detection across cohorts and architectures.

Architecture	PTB-XL knee	F1@knee/F1_max (PTB-XL)	Chapman knee	F1@knee/F1_max (Chapman)
LR	*N* = 4	98.4%	*N* = 3	98.2%
Ridge	*N* = 4	98.4%	*N* = 3	98.2%
LightCNN	*N* = 3	96.3%	*N* = 3	98.3%
Transformer	*N* = 3	96.7%	*N* = 3	97.6%

Chapman knee values are corroborated by both the per-N transfer approach and the full-enumeration approach (where applicable).

Combined with the within-PTB-XL knee analysis (Section 4.3), the data establish two consistencies. Across architectures—linear (LR, Ridge), convolutional (LightCNN), and attention-based (Transformer)—all locate the knee in the *N* = 3–4 band. Across cohorts—PTB-XL and Chapman, two clinical-hospital datasets with different patient demographics and labeling conventions—all four architectures converge to *N* = 3 on Chapman, and the slight PTB-XL → Chapman knee shift for linear models (*N* = 4 → *N* = 3) preserves the recommended *N* = 4 Tier-2 configuration as a high-performing operating point (≥ 98% of per-cohort maximum F1 in both cohorts). We interpret this combined evidence as strong support for the claim that the efficiency–accuracy knee is a stable property of the 12-lead system's information geometry rather than an artefact of any specific architecture or training distribution.

#### 4.8.4 Limits of cross-cohort transfer: configuration- and metric-level dissociations

While the knee location proved cohort-stable, the full Chapman enumeration uncovered two dissociations that qualify the cross-cohort generalisation story and add nuance to the deployment recommendations.

##### 4.8.4.1 Configuration-level instability beyond *N* = 1

Although the N at which performance saturates is cohort-invariant, the specific lead subset achieving that performance is not. Table 11 reports the overlap between PTB-XL and Chapman top-3 best-F1 configurations at each N. At *N* = 1 the two cohorts largely agree (LR: 3 of 3 overlap; Ridge and LightCNN: 2 of 3); from *N* = 2 onwards, the overlap collapses to 0–1 of 3 across all three exhaustively enumerated models.

**Table 11:** PTB-XL ↔ chapman top-3 best-F1 configuration overlap, by model and N.

N	LR	Ridge	LightCNN
1	3/3	2/3	2/3
2	0/3	0/3	0/3
3	1/3	1/3	0/3
4	0/3	0/3	0/3
5	0/3	0/3	0/3
6	0/3	0/3	0/3

Overlap = number of configurations shared between the two cohorts' top-3 lists at the given N.

A representative example: the LR best single lead is AVR on Chapman (Chapman 3-class F1 = 0.649) but II on PTB-XL—a divergence reflecting Chapman's enrichment in arrhythmia presentations, for which AVR's view of the right-atrial activation vector is particularly informative. Similarly, LR's best 4-lead Chapman configuration is II + V1 + V2 + V5 (Chapman F1 = 0.706), whereas its best 4-lead PTB-XL configuration is I + AVF + V2 + V5 (PTB-XL F1 = 0.671); both perform well within their cohort, but exchanging a single limb-lead choice between them costs approximately 2 percentage points of F1.

We interpret this in light of the prevalence shifts documented in Section 4.8.1 (Chapman MI = 4.3% vs. PTB-XL 25.1%; Chapman STTC = 51.6% vs. PTB-XL 24.0%) and the label-mapping heterogeneity inherent in cross-cohort SNOMED-to-superclass mapping. The relative diagnostic value of each lead depends on which morphological abnormalities dominate the cohort: the information-theoretic optimum at a given N (its position relative to the knee) is determined by lead-system structure, but which subset of leads best exploits that information depends on the diagnostic targets actually present.

This has a concrete implication for the deployment recommendations (Section 4.5): the N of recommended Tier-2 (*N* = 4) and Tier-3 (*N* = 7) configurations is a robust population-level default, but the specific lead choice within each tier should ideally be re-derived for a target deployment population if its diagnostic mix differs substantially from PTB-XL's. We update the framing of recommendations in Section 5.4 accordingly to emphasise that the tiers are population-level defaults rather than universal optima.

##### 4.8.4.2 Best-PCC vs. best-F1 dissociation

A second dissociation emerged from Chapman's full enumeration: the configuration with the best reconstruction fidelity (mean PCC) is generally not the configuration with the best downstream diagnostic F1. The most striking instance is LR at *N* = 4 (Table 12): the best-PCC configuration achieves mean PCC = 0.889 with Chapman three-class F1 = 0.691, while the best-F1 configuration achieves PCC = 0.770 with F1 = 0.706. The best-PCC configuration has 15% higher per-lead reconstruction fidelity but 2.2% lower diagnostic accuracy.

**Table 12:** Best-PCC vs. best-F1 dissociation at *N* = 4 (LR, Chapman primary).

Optimisation target	Configuration	Mean PCC	Chapman F1₃	PTB-XL F1₅
Best F1	II + V1 + V2 + V5	0.770	0.706	0.667
Best PCC	III + AVR + V2 + V4	0.889	0.691	0.671

Higher PCC does not imply higher diagnostic F1.

This dissociation is consistent with the broader observation from Section 4.3 that high reconstruction quality does not automatically translate to high diagnostic utility: diagnosis depends on whether the reconstructed signal preserves the specific morphological features the classifier was trained to detect, which aggregate per-lead PCC does not measure. The dissociation is more pronounced on cross-cohort transfer (Chapman) than within-cohort (PTB-XL), suggesting that distributional shift amplifies the gap between reconstruction fidelity and diagnostic discriminability. We interpret this as a methodological caveat: PCC serves as a useful but insufficient proxy for diagnostic value, particularly under distributional shift. We retain PCC in our benchmarking as the canonical reconstruction-quality metric and as a fast screening tool for configuration ranking, but emphasise that joint evaluation with downstream F1—and ideally with cross-cohort F1—is necessary for selecting deployment configurations whose diagnostic utility holds across cohorts.

#### 4.8.5 Implicit domain adaptation from reduced-lead reconstruction

A noteworthy observation arose during external-cohort zero-shot evaluation. For several reduced-lead configurations, F1 on the external cohort exceeded the F1 achieved by direct classification of real 12-lead signals through the frozen ResNet1D. On Chapman, the V6 reconstruction achieved three-class macro-F1 = 0.756 (LR; 99% retention of within-PTB-XL F1), compared to 0.706 (LightCNN direct 12-lead, 93% retention); a smaller but consistent effect was observed for the II single-lead configuration on CPSC (LR retention 102.7%) and Chapman (LR retention 105.8%), and on the V6 reconstruction on Chapman across all linear models. The effect is most pronounced on Chapman but is also present in a subset of CPSC configurations, with magnitude correlated with cohort distributional proximity to PTB-XL.

We interpret this as an implicit domain adaptation phenomenon. The reconstruction models, having been trained on PTB-XL, generate output signals that retain PTB-XL's distributional characteristics—waveform morphologies, baseline filtering effects, and inter-lead amplitude relationships—to which the frozen classifier is calibrated. When the input cohort shares acquisition and labeling characteristics with the training cohort, this implicit alignment partially compensates the residual distributional shift, yielding modest but consistent improvements over raw external-cohort classification. The effect is stronger on Chapman than on CPSC because Chapman shares more acquisition and labeling commonalities with PTB-XL, allowing the reconstruction's PTB-XL prior to align more usefully with the target distribution; the smaller CPSC effect indicates the same mechanism in attenuated form rather than absence.

We do not treat this as a clinical recommendation in itself: reconstructed signals may smooth cohort-specific morphological variation that could be diagnostically relevant in other settings, and the magnitude of the effect is modest (a few percentage points of macro-F1). We document the observation as a potentially useful property of reduced-lead reconstruction pipelines for cross-cohort deployment, and leave its systematic characterisation—including whether the effect generalises across other distributional-shift regimes—to future work.

#### 4.8.6 Per-class retention and limitations

While aggregate three-class retention is strong on both external cohorts, per-class F1 retention varies. On Chapman primary, NORM and STTC retain comparably to the aggregate macro-F1, and CD retains within roughly 0.50–0.58. MI and HYP show weaker per-class retention (F1 ≈ 0.28–0.33 on Chapman versus 0.50–0.70 on PTB-XL); CPSC has no labeled MI or HYP and cannot evaluate these classes at all.

Two factors drive the MI/HYP gap:

- *Prevalence shift.* Chapman MI prevalence is 4.3%, vs. 25.1% in PTB-XL; HYP is 23.5% in Chapman vs. 12.2% in PTB-XL (driven by Chapman's inclusion of LVH-by-voltage criterion as a HYP-mapped code). Both directions of prevalence shift induce prior-shift effects on F1: a classifier calibrated to higher MI prevalence will over-predict MI on a low-prevalence cohort, depressing precision.
- *Label-mapping heterogeneity.* SNOMED-CT codes do not map uniquely to PTB-XL superclasses; the mapping in Supplementary Table S3 reflects expert judgement supplemented by the CinC 2021 dx_mapping reference, but residual definitional differences (for example, Chapman's poor-R-wave-progression coded as STTC, abnormal Q-wave as MI) introduce non-reconstruction-related variance in per-class F1.

We disclose both factors as inherent limitations of cross-cohort external validation under prevalence and labeling shift rather than as reconstruction-pipeline failures (see Section 5.5). The aggregate three-class macro-F1 retention reported in Table 9, computed on the classes with the most consistent cross-cohort definition, provides the most reliable summary of deployment-configuration generalisation.

### 4.9 Frozen classifier sensitivity control

A potential concern with our evaluation framework is that the downstream ResNet1D classifier was trained and frozen on real 12-lead signals, then evaluated on reconstructed signals from reduced-lead inputs. If the frozen classifier systematically penalises reconstructed signals whose morphology differs subtly from the training distribution, the absolute F1 values and the location of the efficiency–accuracy knee could be artefacts of this frozen-classifier design rather than properties of the lead system itself. To address this concern, we performed a fine-tuning sensitivity analysis on two representative configurations.

#### 4.9.1 Experimental design

For each of two representative configurations—*N* = 4 (I + II + AVR + AVF, the Tier-2 recommendation) and *N* = 7 (I + II + III + AVR + AVL + AVF + V1, the Tier-3 recommendation)—we generated reconstructed 12-lead signals on the PTB-XL training set using LightCNN, then fine-tuned the ResNet1D classifier's final fully-connected layer on these reconstructed signals: training proceeded for 10 epochs at learning rate 1 × 10⁻⁴ and batch size 256, with early stopping on validation macro-F1. Performance was then re-evaluated on the PTB-XL test set with the fine-tuned classifier and the same N-lead reconstructed input.

#### 4.9.2 Results

Fine-tuning produced only marginal changes in macro-F1 (Table 13). At *N* = 4, *Δ*F1_finetune =  + 0.0006—approximately one fiftieth of the cross-N marginal gain at the same step (*Δ*F1 = 0.031 from *N* = 3 → *N* = 4). At *N* = 7, *Δ*F1_finetune = −0.0033, slightly negative, consistent with mild overfitting to the fine-tuning subset, and again far smaller in magnitude than the cross-N marginal gain in the saturation regime (*Δ*F1 ≈ 0.002 from *N* = 6 → *N* = 7).

**Table 13:** Frozen-classifier sensitivity control: macro-F1 with frozen vs. fine-tuned classifier at two representative configurations.

Configuration	F1 (frozen)	F1 (fine-tuned)	*Δ*F1_finetune	*Δ*F1_cross-N
*N* = 4: I + II + AVR + AVF	0.6286	0.6292	+0.0006	+0.031
*N* = 7: 7-lead set	0.6532	0.6499	−0.0033	+0.002

The change *Δ*F1_finetune is far smaller than the cross-N marginal gain *Δ*F1_cross-N at the same N step, indicating that the frozen-classifier paradigm does not distort knee localisation.

#### 4.9.3 Interpretation

The frozen-classifier paradigm is intentional and reflects the realistic deployment scenario in which a diagnostic algorithm is pre-trained on a large reference dataset and then deployed without per-device re-training. The sensitivity control confirms that this paradigm does not bias the knee position: fine-tuning recovers at most six thousandths of additional F1, two orders of magnitude smaller than the *Δ*F1 between adjacent N values in the rising portion of the curve and comparable in magnitude to the per-class measurement noise.

This conclusion is further supported by the cross-cohort finding in Section 4.8.5: on external cohorts, the frozen classifier actually favours reconstructed signals over real 12-lead signals for several configurations, the opposite of the concern raised by the frozen-classifier critique. Together, the within-cohort fine-tuning analysis and the cross-cohort domain-adaptation observation indicate that the choice of a frozen classifier is methodologically sound and that observed retention values reflect genuine reduced-lead diagnostic capability rather than classifier-specific biases.

## 5 Discussion

### 5.1 Interpretation of the efficiency–accuracy knee

The finding that diagnostic accuracy saturates near *N* = 4 is consistent with the mutual information structure of the 12-lead system. Specifically, *N* = 4 leads capture 93.5% of the 12-lead F1 upper bound on average, *N* = 6 leads capture 97.6%, and *N* ≥ 7 contributes less than 0.007 absolute F1 per additional lead. This steep-then-flat profile arises because the dominant independent information dimensions in the 12-lead system—the frontal plane (spanned by the four-electrode limb bundle) and the two most informative transverse positions—are captured within the first 4–6 well-chosen leads. The remaining leads contribute largely redundant spatial sampling.

The knee location is robust across both architecture and cohort axes. Across four reconstruction architectures spanning the linear–nonlinear and convolutional–attention spectrum (LR, Ridge, LightCNN, Transformer), Kneedle places the knee within the narrow *N* = 3–4 band, and at the recommended *N* = 4 each architecture retains ≥ 98% of its per-architecture F1 maximum (Section 4.3). The one-lead discrepancy between linear and nonlinear models (LR/Ridge knee *N* = 4 vs. LightCNN/Transformer knee *N* = 3) reflects the nonlinear models' substantially higher *N* = 1 PCC (0.765–0.783 vs. 0.533), which compresses the effective gain range of the N–F1 curve and shifts the Kneedle-detected normalised knee one step earlier—a normalisation-side artefact of inflection detection rather than evidence of architecture-specific structure. Across cohorts, on Chapman-Shaoxing all four architectures converge to *N* = 3, within one lead of the PTB-XL knee, and full *N* = 1–6 enumeration on Chapman corroborates that F1 saturates by *N* = 3 for all three exhaustively enumerated models (Section 4.8.3). We therefore interpret the efficiency–accuracy knee as a stable property of the 12-lead system's information geometry, not an artefact of reconstruction architecture or training cohort.

Within this stable knee location, two finer-grained nuances are worth noting. First, the nonlinear models achieve higher PCC than the linear baselines at small N (*Δ*PCC ≈ 0.23 at *N* = 1), confirming the value of nonlinear inference when the reconstruction task is most under-determined; however, the corresponding F1 gap at *N* = 1 is modest (≤ 0.032), indicating that diagnostic classification is largely robust to the specific reconstruction architecture even at minimum coverage. At *N* ≥ 5, all four architectures converge to within 0.005 F1 of one another, suggesting that with sufficient input the linear structure of the lead system fully determines reconstruction quality. Second, while the N at which performance saturates is cohort-stable, the specific lead subsets achieving that performance are not (Section 4.8.4): cross-cohort overlap between PTB-XL and Chapman top-3 configurations drops to 0–1 of 3 from *N* = 2 onwards, reflecting cohort-specific differences in diagnostic-target prevalence and label structure. This separation between the cohort-invariant knee location and the cohort-dependent specific configurations underpins the population-level-defaults framing of the deployment recommendations developed in Section 5.4.

The clinical adequacy of the recommended configurations depends on the deployment setting and is not a single threshold. For triage and screening applications—the Tier-1 (V6, macro-F1 = 0.509) and Tier-2 (I + II + AVR + AVF, macro-F1 = 0.616) recommendations—the relevant comparator is the current generation of FDA-cleared single- and few-lead consumer and clinical wearables, whose reported accuracies for general arrhythmia and ST-segment screening fall in a comparable or lower range (1–3, 6). For applications requiring full diagnostic certainty (definitive STEMI confirmation, comprehensive multi-class diagnosis), the 12-lead standard remains the reference; the Tier-3 7-lead set (macro-F1 = 0.657, retaining 97.5% of the 12-lead F1) approaches but does not replace this reference. We therefore frame the three tiers explicitly as complements to, rather than replacements for, the 12-lead standard, each suited to a different point on the accuracy–accessibility trade-off; this framing is developed further in Section 5.4.

### 5.2 The electrode-contact model: mathematical and clinical interpretation

#### 5.2.1 Mathematical perspective

The CLS objective CLS(C, α) = α · (F1/F1_upper) + (1−α) · (1−E_norm) is a convex combination of two normalised scalars, yielding a score on [0, 1]. Its key mathematical property is the non-linear mapping from N to E introduced by the limb-electrode sharing constraint:

E(C)=4⋅1[∃limblead∈C]+|precordial∩C|

This creates a discrete jump: any configuration containing at least one limb lead pays a fixed cost of 4, regardless of how many limb leads are included. Consequently, the marginal electrode cost of transitioning from one to six limb leads is zero—while the F1 gain from adding limb leads is positive but diminishing. Mathematically, this means the gradient of CLS with respect to the number of limb leads is always positive (adding limb leads improves F1 without worsening the efficiency term), generating a natural incentive to exhaust the limb-electrode bundle before introducing any precordial leads. The CLS landscape therefore contains a plateau region at E = 4 where all-limb configurations dominate under moderate efficiency weights, and a ridge of mixed limb-precordial configurations that becomes optimal only when the F1 gain from adding one precordial contact justifies the normalised cost penalty of 1/9 ≈ 0.111 in the efficiency term.

The α threshold at which the globally optimal combination transitions from a single precordial lead (E = 1) to a mixed configuration can be derived analytically. For the single-lead V6 configuration we observe F1₁ = 0.509 (Table 4), and for the most accuracy-favourable 5-lead configuration (I + III + AVR + AVF + V2 at E = 5) we observe F1₅ = 0.659. Setting the two CLS expressions equal,

$$\mathrm{CL}S_{\mathrm{single}}=\alpha \cdot \frac{F_{1,1}}{0.6751}+(1-\alpha )\cdot \frac{8}{9}$$

$$\mathrm{CL}S_{\mathrm{mixed}}=\alpha \cdot \frac{F_{1,5}}{0.6751}+(1-\alpha )\cdot \frac{4}{9}$$

and solving for α yields:

$$\alpha^{*}=\frac{4/9}{(F_{1,5}-F_{1,1})/0.6751+4/9}=\frac{4/9}{(0.659-0.509)/0.6751+4/9}\approx 0.63$$

This α* ≈ 0.63 matches the empirical crossover observed between α = 0.50 and α = 0.65 in Table 4, providing a closed-form validation of the sensitivity analysis. An analogous derivation for the V6 → V2 + V6 transition at α* ≈ 0.564 and the V6 → 5-lead transition at α* ≈ 0.667 (both observed in Table 4's empirical scan) is provided in Supplementary Material.

#### 5.2.2 Medical and biological perspective

The electrode-contact model has a direct grounding in cardiac electrophysiology. The six limb leads are not six independent measurements but six linear combinations of three independent limb potentials (Einthoven's law: I−II + III = 0). Physically, all six limb leads are derived from the same three active electrode potentials, which explains why the fixed-cost model is biologically correct. The information-theoretic consequence is that the six limb leads together span only a 2-dimensional subspace of cardiac electrical activity—the frontal plane projection of the cardiac dipole. This is why all-limb configurations plateau in F1 at approximately 0.61: they comprehensively sample the frontal plane but are blind to the transverse plane information encoded in the precordial leads.

The precordial leads V1–V6 sample the transverse (horizontal) plane at six positions from right-septal (V1) to left-lateral (V6). Each represents a genuinely new spatial dimension not captured by the limb circuit. The empirical finding that V2 and V5 appear most frequently in top-ranked mixed configurations is consistent with their known clinical importance: V2 is primary for detecting right ventricular and septal pathology (including right bundle-branch block, anterior MI), while V5 is most sensitive for left ventricular lateral wall ischaemia. These leads maximise the mutual information between the observed subset and the withheld 12-lead signal precisely because they capture spatial directions orthogonal to both the frontal-plane limb lead bundle and each other.

The asymmetry between F1-based and PCC-based CLS recommendations (Tables 4 vs. 5) has a physiological explanation: PCC is maximised by configurations that best reconstruct the morphological waveform (amplitude and timing of P, QRS, T waves), while macro-F1 is maximised by configurations whose reconstructed 12-lead ECG preserves the diagnostic features that the frozen ResNet1D classifier was trained to detect. These are not identical objectives: a configuration could achieve high PCC by faithfully reconstructing the overall waveform envelope while missing a subtle 0.1 mV ST elevation that determines the MI classification.

The electrode-contact cost model E(C) thus has three layers of grounding that, taken together, justify its use as the efficiency component of the Composite Lead Score. Derivation: the model follows directly from Einthoven's law (which constrains the six limb leads to a two-dimensional subspace determined by three independent limb potentials) combined with the physical fact that each precordial lead requires an independent chest-electrode placement. Both contributions enter the cost expression as direct counts—four limb-bundle contacts when any limb-derived lead is required, plus one contact per included precordial lead—without free parameters. Use: within the CLS objective, E(C) provides a clinically interpretable efficiency penalty whose unit (electrode contacts) maps directly to device-design decisions (number of electrodes, preparation time, patient burden) and avoids the systematic mis-ranking that arises when lead count N is used as a naive efficiency proxy (Section 4.6). Diagnostic insight: the resulting CLS landscape contains a plateau region at E = 4 where all-limb configurations dominate under moderate efficiency weights and a ridge of mixed limb-precordial configurations that becomes optimal only when the F1 gain from adding a precordial contact justifies the normalised cost penalty of 1/9 ≈ 0.111 in the efficiency term. The two complementary perspectives—the lead-count CLS_N and the electrode-contact CLS_E—are compared head-to-head in Section 4.6 and yield qualitatively different deployment recommendations specifically in the *N* = 4–6 range, where the limb-electrode-sharing effect is most consequential.

### 5.3 Sensitivity analysis and recommendation stability

**Figure 10:**
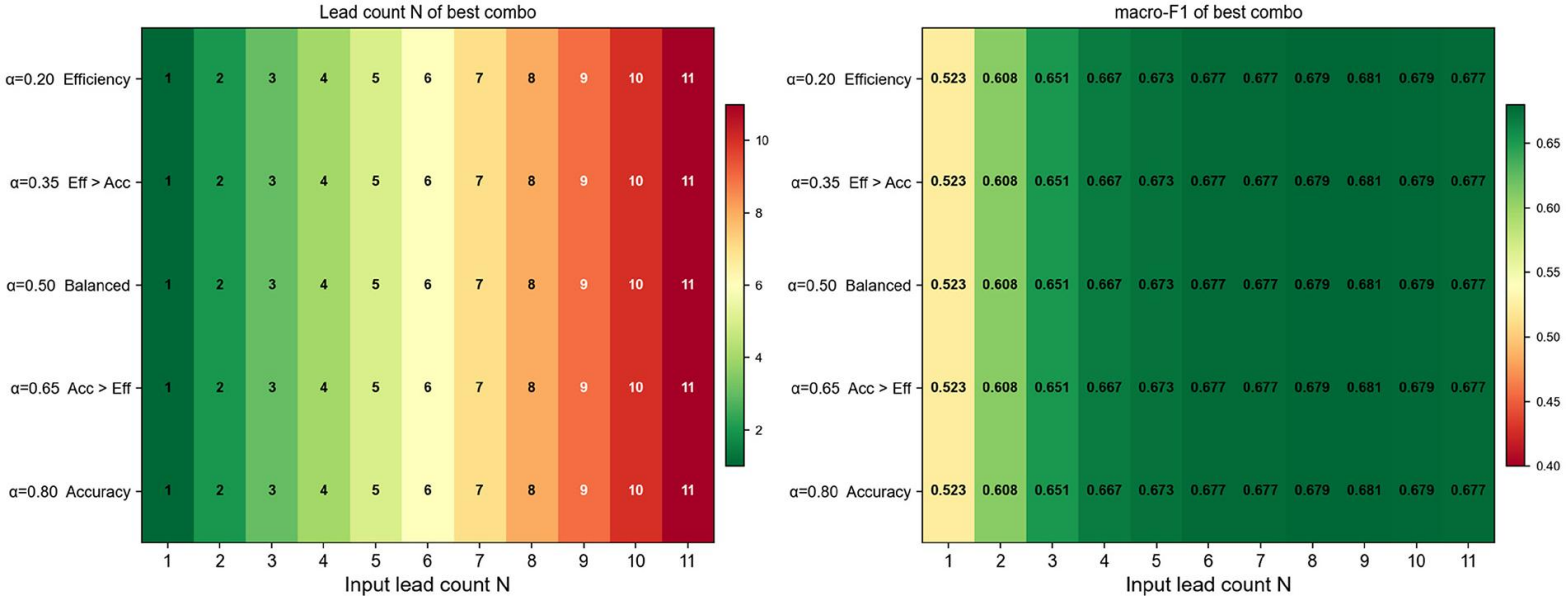
N-based sensitivity heat maps (CLS_N_F1). Left: lead count N of per-N per-*α* optimal combination. Right: macro-F1. Compare with Figure 6 (E-based): divergence is largest at *N* = 4–6, where E-based selects all-limb configurations (E = 4, lower F1) and N-based selects mixed limb-precordial configurations (higher F1).

The five-point CLS sensitivity analysis (Tables 4, 5, Figures 6, 10) reveals that the efficiency-dominant regime (α ≤ 0.50) consistently recommends single-lead precordial configurations (V5 or V6, E = 1), while the accuracy-dominant regime (α ≥ 0.65) recommends small mixed sets of *N* = 2–5 leads with E = 2–6 contacts. These regimes are separated by a well-defined crossover near α = 0.63 (derived analytically in Section 5.2.1). Configurations in the *N* = 4–6 range with all-limb composition (E = 4) represent a robust intermediate optimum: they appear in the top 3 under all five weight profiles for the E-based metric, because they exploit the limb-electrode sharing property to achieve F1 ≈ 0.61–0.66 with only four electrode contacts. This combination does not appear at all in the N-based top recommendations (Section 4.6), underscoring that the choice of efficiency metric—not just the accuracy weight—substantially shapes the clinical guidance.

A complementary perturbation of the cost model itself, in which the equal-weight assumption across V1–V6 is replaced by faster-placement weights for V3/V4 (0.8) and slower-placement weights for V1/V2 (1.3), produces no rank reordering among the top-3 deployment recommendations at any α (Supplementary Table S5). Notably, the specific lead choice at α = 0.80 shifts between Scheme A and Baseline (I + AVR + AVF + V3 + V5 vs. I + III + AVR + AVF + V2), consistent with the cross-cohort observation in Section 4.8.4 that within-tier optimal configurations are sensitive to parameterisation while the N level is stable. This indicates that the recommendations are robust not only to accuracy-efficiency weighting α but also to plausible perturbations of the cost-model parameterisation itself.

### 5.4 Clinical implications

The deployment configurations identified in Section 4.5 are positioned as population-level defaults, derived from PTB-XL and externally validated on CPSC2018 and Chapman-Shaoxing (Section 4.8): they specify, for each of three deployment tiers, an evidence-based lead count and a representative configuration whose diagnostic performance has been verified to transfer with ≥ 83% retention to two independent external cohorts spanning different degrees of distributional shift. Within each tier, the recommendation should be interpreted as a population-level default rather than a universal optimum, as Section 4.8.4 demonstrates that the specific optimal lead subset at a given N can differ between cohorts when the underlying diagnostic-target prevalence differs substantially.

For pre-hospital emergency deployment (time-critical, minimal setup), a single-chest-electrode configuration—Tier 1: V6, E = 1, macro-F1 = 0.509—provides a viable triage screen when paired with a lightweight reconstruction model; the single-electrode patch can be applied in under five seconds. For community screening programmes (balanced accuracy and burden), an all-limb configuration—Tier 2: I + II + AVR + AVF, *N* = 4, E = 4 contacts, macro-F1 = 0.616—requires only wrist and ankle clips with no chest preparation and achieves 91.5% of the real 12-lead F1. For home monitoring (near-full diagnostic value), the Tier-3 recommendation extends the all-limb bundle by adding a single precordial contact at V1. The resulting seven-lead configuration (I + II + III + AVR + AVL + AVF + V1) is acquired using only five electrodes: the four limb electrodes (RA, LA, LL, RL) that jointly produce all six limb leads via Einthoven's law, plus the V1 chest electrode. This five-electrode setup attains macro-F1 = 0.657—97.5% of the real 12-lead F1—and is therefore a practical configuration for extended wearable use, where the modest additional burden of one chest electrode over the all-limb Tier-2 set is justified by the substantial gain in transverse-plane diagnostic coverage.

Importantly, the disease-subgroup analysis (Section 4.7) shows that MI detection remains the most lead-hungry task: reliable MI diagnosis requires at least one precordial lead to capture anterior and lateral ST-segment changes. The per-class F1 data from Table 7 quantify this precisely. At *N* = 1 (lead II only), MI classification achieves F1 = 0.313, barely above chance for a 25%-prevalence class. Adding a single precordial lead to form a 2-lead set (AVR + AVF) raises MI F1 to 0.533, an absolute gain of +0.220—the single largest per-class improvement at any step in the entire benchmark. The critical transition is at *N* = 3 (II + AVR + V2), where MI F1 reaches 0.599 and the gap to the 12-lead upper bound narrows to 0.064. In contrast, NORM detection is largely resolved at *N* = 1 (F1 = 0.791), confirming that a single-lead device is adequate for ruling out gross abnormality. These findings support a tiered clinical guideline: for NORM/CD screening, *N* = 1–2 is sufficient; for MI triage, a minimum of *N* = 2 with at least one precordial lead is required (recommended: AVR + V2); for comprehensive multi-class diagnosis including HYP, *N* = 5–6 covering both frontal and transverse planes is the practical minimum.

#### 5.4.1 Caveat on within-tier specific lead choice

The recommendations above identify the N and the population-level default configuration at each tier. Where a deployment population differs substantially from PTB-XL in diagnostic-mix (for example, a screening programme dominated by arrhythmia rather than morphology), the specific lead choice within a tier may benefit from cohort-specific re-derivation. Concretely, our Chapman-Shaoxing enumeration (Section 4.8.4) finds that the best *N* = 1 lead on that arrhythmia-enriched cohort is AVR rather than the PTB-XL-optimal V6, reflecting Chapman's prevalence of right-atrial activation pathologies. We therefore recommend that, in distributionally distinct target populations, the population-level configurations be re-evaluated against local pilot data before being adopted as final device specifications. The within-tier N is robust; the within-tier specific leads are an optimisation that can be re-run quickly on cohort-specific data using the publicly released benchmark code.

### 5.5 Limitations

Several limitations remain. First, although external zero-shot validation on CPSC2018 and Chapman-Shaoxing demonstrates retention ≥ 83% of within-PTB-XL three-class F1 (Section 4.8), broader validation across geographically and demographically diverse cohorts—particularly populations with non-European-ancestry electrocardiographic norms and with markedly different age and pathology distributions—remains an open need. Second, we treat lead selection as a static pre-assignment problem; real wearable devices may have mechanical constraints on electrode placement that further restrict available configurations, and dynamic lead-selection frameworks adapting in real time based on initial signal quality were not evaluated. Third, although a complementary sensitivity analysis confirms recommendations are stable under perturbations of precordial electrode cost (Section 5.3, Supplementary Table S5), the equal-weight assumption among V1–V6 in the primary CLS analysis remains a simplification, and a refined model based on quantified clinical placement-time data may sharpen the pre-hospital deployment recommendations. Fourth, although Section 4.8.4 demonstrates that the N of the efficiency–accuracy knee is cohort-stable, the specific lead subset optimal within each N can differ across cohorts (PTB-XL ↔ Chapman top-3 overlap drops to 0–1 of 3 from *N* = 2 onwards), implying that population-level configurations should be re-derived on cohort-specific data when deploying to populations whose diagnostic mix differs substantially from PTB-XL's. Fifth, while we evaluate four model families covering the linear-through-attention spectrum, emerging architectures such as diffusion-model-based reconstruction (13) and structured state-space sequence models (24) may yet exhibit qualitatively different saturation patterns in the severely under-determined regime (*N* = 1–2). Finally, the downstream classifier was held frozen throughout to model realistic deployment conditions; Section 4.9 verifies that fine-tuning the classifier on reconstructed signals shifts F1 by less than 0.004, confirming that the frozen-classifier paradigm does not bias knee localisation, but does not rule out larger effects under classifier architectures other than ResNet1D.

### 5.6 Future work

Several directions merit further investigation. First, personalised lead selection based on patient-specific factors (age, sex, body habitus, known pathology) may outperform the population-level configurations identified here; integration with clinical metadata would extend our benchmark from population-level defaults to patient-level optimal sets. Second, the static lead assignment model assumed throughout should be evaluated against dynamic selection frameworks, where the active lead set is adapted in real time based on initial signal quality and preliminary classification confidence. Third, cohort-specific within-tier re-derivation of the optimal lead subset—using the same exhaustive-enumeration methodology applied here, but on a target deployment population—would convert the population-level defaults into population-specific recommendations; our Chapman-Shaoxing enumeration (Section 4.8.4) provides a worked example. Fourth, emerging architectures—in particular diffusion-model-based signal synthesis (13) and structured state-space sequence models such as Mamba (24)—may exhibit qualitatively different saturation patterns in the severely under-determined regime (*N* = 1–2) and warrant systematic comparison against the four architectures evaluated here. Fifth, a refined electrode-contact cost model incorporating empirical time-to-place data and patient-specific factors (motion artefact, skin condition, obesity) would sharpen the pre-hospital deployment recommendations beyond the perturbation analysis in Section 5.3. Finally, hardware co-design—jointly optimising the physical sensor form factor with the lead selection model—represents the ultimate translational step for this line of research (25).

## 6 Conclusion

This paper establishes the first exhaustive, model-agnostic benchmark for reduced-lead 12-lead ECG reconstruction, systematically evaluating all 4,094 C(12, N) lead configurations across *N* = 1*–*11 under four reconstruction families and a three-axis evaluation framework. The central empirical finding is that the efficiency–accuracy trade-off contains a sharp, model-consistent inflection point at *N* = 4: four-lead configurations capture 93.5% of 12-lead diagnostic accuracy on average, six-lead configurations capture 97.6%, and each additional lead beyond *N* = 7 contributes less than 0.7 percentage points of F1—making further expansion of low marginal value for most deployment contexts. A novel electrode-contact cost model formalises the asymmetric operational burden of limb vs. precordial lead addition, enabling the Composite Lead Score (CLS) to serve as a unified, clinically interpretable objective that translates multi-axis performance trade-offs into a single deployable design criterion. Five-point sensitivity analysis confirms that these recommendations remain stable: the all-limb 4-lead set (I + II + AVR + AVF, E = 4 contacts) is robustly optimal across all tested accuracy-efficiency weightings under the electrode-contact metric, while single-chest-electrode configurations dominate only when efficiency is weighted overwhelmingly above accuracy.

For practitioners, these results translate directly into three evidence-based hardware targets, externally validated on two independent cohorts: a single-contact precordial patch (V6, F1 = 0.509) for rapid pre-hospital triage, the all-limb 4-lead set (I + II + AVR + AVF, F1 = 0.616) for community screening, and a 7-lead mixed configuration at 5 electrode contacts (F1 = 0.657) for sustained home monitoring. Zero-shot external validation on CPSC2018 and Chapman-Shaoxing confirms ≥ 83% three-class F1 retention for all three configurations across both cohorts, and an exhaustive replication on Chapman-Shaoxing demonstrates that the *N* = 3–4 knee location is consistent across all four reconstruction architectures evaluated (linear, ridge, convolutional, Transformer) and across both cohorts. A numerical reference table of optimal configurations for *N* = 1–8, including disease-specific recommendations, is provided in Supplementary Tables S1a–c, S2 (Supplementary Material). Within each deployment tier, we position our recommendations as population-level defaults: the N is cohort-stable, but the specific optimal lead subset at a given N can benefit from cohort-specific re-derivation in distributionally distinct deployment populations. These findings provide a principled, data-driven foundation for the design and clinical guideline development of next-generation portable ECG devices, and establish a reproducible benchmark against which future personalised and adaptive reconstruction methods can be evaluated.

## Data Availability

The original contributions presented in the study are included in the article/Supplementary Material. The complete analysis code, per-combination benchmark results, and reproducibility scripts are publicly available at: https://github.com/ZJUXiaoyu/ecg-lead-selection-benchmark. Further inquiries can be directed to the corresponding author.
