# Supplementary material for: Systematic benchmark of reduced-lead configurations for 12-lead ECG reconstruction: multi-model evaluation across all possible subsets
Source: Front Cardiovasc Med. 2026 Jun 29;13:1856211. doi: 10.3389/fcvm.2026.1856211 (PMC13357747; doi:10.3389/fcvm.2026.1856211)
Supplement: Supplementary file 1 [file Datasheet1.docx]

**Supplementary Materials**

**
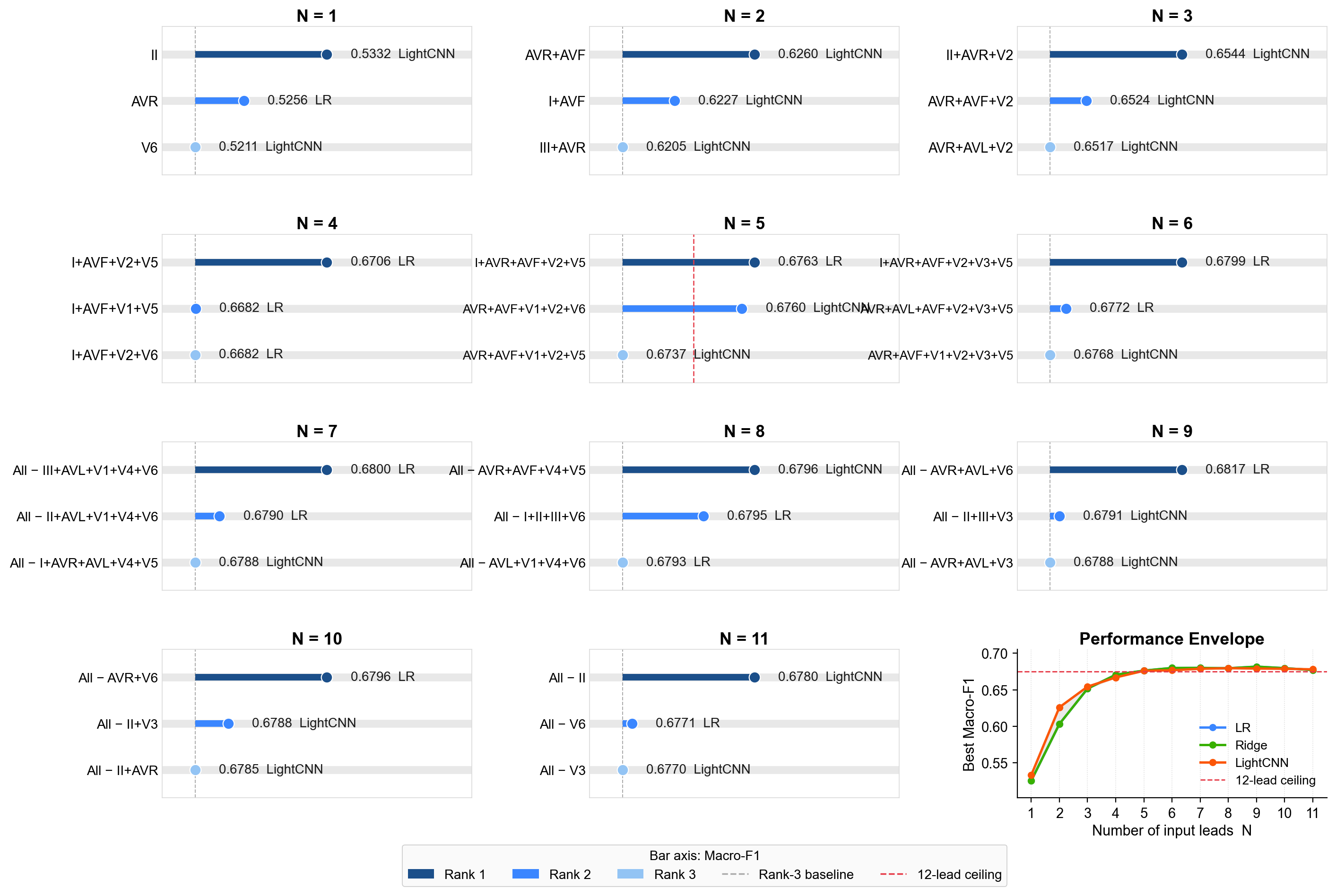
**

Figure S1. Top-3 lead configurations per input count N ranked by macro-F1 (LightCNN / LR / Ridge). Each panel (N = 1–11) shows a lollipop chart: the horizontal stem extends from the Rank-3 value (grey dashed baseline) to each configuration's macro-F1, making within-N differences directly readable. Rank 1 / 2 / 3 are encoded in dark-to-light blue. Value labels show 4-decimal macro-F1 and the model achieving it. Panel 12: performance envelope (best macro-F1 per N per model) with inter-model spread shading and 12-lead ceiling (red dashed, F1 = 0.6751).


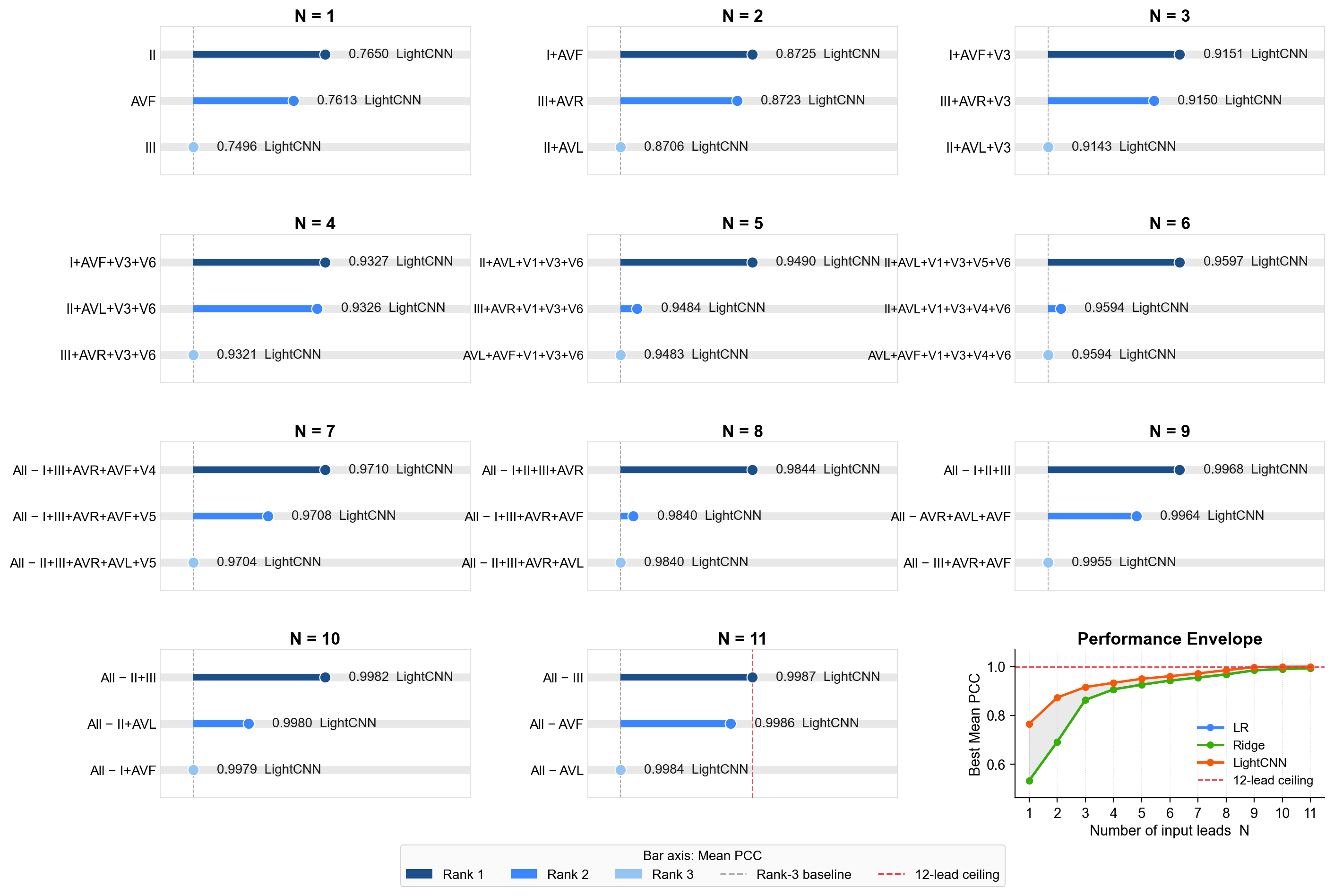


Figure S2. Top-3 lead configurations per input count N ranked by mean PCC (LightCNN only, all N). Layout identical to Figure S1. Each panel shows the three lead subsets with the highest mean Pearson Correlation Coefficient (evaluated on withheld 12 − N leads). Panel 12: performance envelope for mean PCC; 12-lead ceiling computed as the maximum mean PCC achieved at N = 11 (0.9987).

Table S1a. Top-3 lead configurations per input count N ranked by reconstruction fidelity (Mean PCC). For each N, the three highest-PCC configurations are listed in descending order (Rank 1–3). E = electrode contact count (1–10). Macro-F1 and CLS are provided for cross-metric reference. All metric values are the best achievable across LR, Ridge, and LightCNN. For N ≥ 9 the configuration is written as All−{excluded leads} for brevity. ★ marks the Kneedle-detected efficiency–accuracy knee (N = 4).

| **N** | **Rank** | **Configuration** | **E** | **Mean PCC** | **Macro-F1** | **CLS (α = 0.50)** |
| --- | --- | --- | --- | --- | --- | --- |
| 1 | 1 | **II** | 4 | **0.7650** | 0.5332 | 0.7282 |
|  | 2 | AVF | 4 | 0.7613 | 0.4874 | 0.6943 |
|  | 3 | III | 4 | 0.7496 | 0.4463 | 0.6639 |
| 2 | 1 | **I+AVF** | 4 | **0.8725** | 0.6227 | 0.7945 |
|  | 2 | III+AVR | 4 | 0.8723 | 0.6205 | 0.7929 |
|  | 3 | II+AVL | 4 | 0.8706 | 0.6045 | 0.7811 |
| 3 | 1 | **I+AVF+V3** | 5 | **0.9151** | 0.6382 | 0.7504 |
|  | 2 | III+AVR+V3 | 5 | 0.9150 | 0.6450 | 0.7555 |
|  | 3 | II+AVL+V3 | 5 | 0.9143 | 0.6406 | 0.7523 |
| ★4 | 1 | **I+AVF+V3+V6** | 6 | **0.9327** | 0.6595 | 0.7107 |
|  | 2 | II+AVL+V3+V6 | 6 | 0.9326 | 0.6554 | 0.7076 |
|  | 3 | III+AVR+V3+V6 | 6 | 0.9321 | 0.6562 | 0.7082 |
| 5 | 1 | **II+AVL+V1+V3+V6** | 7 | **0.9490** | 0.6619 | 0.6569 |
|  | 2 | III+AVR+V1+V3+V6 | 7 | 0.9484 | 0.6687 | 0.6619 |
|  | 3 | AVL+AVF+V1+V3+V6 | 7 | 0.9483 | 0.6659 | 0.6598 |
| 6 | 1 | **II+AVL+V1+V3+V5+V6** | 8 | **0.9597** | 0.6670 | 0.6051 |
|  | 2 | II+AVL+V1+V3+V4+V6 | 8 | 0.9594 | 0.6646 | 0.6033 |
|  | 3 | AVL+AVF+V1+V3+V4+V6 | 8 | 0.9594 | 0.6678 | 0.6057 |
| 7 | 1 | **II+AVL+V1+V2+V3+V5+V6** | 9 | **0.9710** | 0.6735 | 0.5544 |
|  | 2 | II+AVL+V1+V2+V3+V4+V6 | 9 | 0.9708 | 0.6728 | 0.5539 |
|  | 3 | I+AVF+V1+V2+V3+V4+V6 | 9 | 0.9704 | 0.6706 | 0.5522 |
| 8 | 1 | **AVL+AVF+V1+V2+V3+V4+V5+V6** | 10 | **0.9844** | 0.6725 | 0.4980 |
|  | 2 | II+AVL+V1+V2+V3+V4+V5+V6 | 10 | 0.9840 | 0.6700 | 0.4962 |
|  | 3 | I+AVF+V1+V2+V3+V4+V5+V6 | 10 | 0.9840 | 0.6734 | 0.4988 |

Table S1b. Top-3 lead configurations per input count N ranked by downstream diagnostic accuracy (Macro-F1). The 12-lead classifier upper bound is F1 = 0.6751. E = electrode contact count. Mean PCC and CLS are provided for cross-metric reference. ★ marks the Kneedle-detected efficiency–accuracy knee (N = 4).

| **N** | **Rank** | **Configuration** | **E** | **Mean PCC** | **Macro-F1** | **CLS (α = 0.50)** |
| --- | --- | --- | --- | --- | --- | --- |
| 1 | 1 | **II** | 4 | 0.7650 | **0.5332** | 0.7282 |
|  | 2 | AVR | 4 | 0.7286 | 0.5256 | 0.7226 |
|  | 3 | V6 | 1 | 0.7101 | 0.5211 | 0.8859 |
| 2 | 1 | **AVR+AVF** | 4 | 0.8688 | **0.6260** | 0.7970 |
|  | 2 | I+AVF | 4 | 0.8725 | 0.6227 | 0.7945 |
|  | 3 | III+AVR | 4 | 0.8723 | 0.6205 | 0.7929 |
| 3 | 1 | **II+AVR+V2** | 5 | 0.8999 | **0.6544** | 0.7624 |
|  | 2 | AVR+AVF+V2 | 5 | 0.9072 | 0.6524 | 0.7610 |
|  | 3 | AVR+AVL+V2 | 5 | 0.9076 | 0.6517 | 0.7604 |
| ★4 | 1 | **I+AVF+V2+V5** | 6 | 0.9308 | **0.6706** | 0.7189 |
|  | 2 | I+AVF+V1+V5 | 6 | 0.9228 | 0.6682 | 0.7171 |
|  | 3 | I+AVF+V2+V6 | 6 | 0.9301 | 0.6682 | 0.7171 |
| 5 | 1 | **I+AVR+AVF+V2+V5** | 6 | 0.9287 | **0.6763** | 0.7231 |
|  | 2 | AVR+AVF+V1+V2+V6 | 7 | 0.9370 | 0.6760 | 0.6674 |
|  | 3 | AVR+AVF+V1+V2+V5 | 7 | 0.9368 | 0.6737 | 0.6657 |
| 6 | 1 | **I+AVR+AVF+V2+V3+V5** | 7 | 0.9394 | **0.6799** | 0.6702 |
|  | 2 | AVR+AVL+AVF+V2+V3+V5 | 7 | 0.9410 | 0.6772 | 0.6682 |
|  | 3 | AVR+AVF+V1+V2+V3+V5 | 8 | 0.9497 | 0.6768 | 0.6124 |
| 7 | 1 | **I+II+AVR+AVF+V2+V3+V5** | 7 | 0.9306 | **0.6800** | 0.6703 |
|  | 2 | I+III+AVR+AVF+V2+V3+V5 | 7 | 0.9312 | 0.6790 | 0.6696 |
|  | 3 | II+III+AVF+V1+V2+V3+V6 | 8 | 0.9545 | 0.6788 | 0.6138 |
| 8 | 1 | **I+II+III+AVL+V1+V2+V3+V6** | 8 | 0.9508 | **0.6796** | 0.6145 |
|  | 2 | AVR+AVL+AVF+V1+V2+V3+V4+V5 | 9 | 0.9654 | 0.6795 | 0.5588 |
|  | 3 | I+II+III+AVR+AVF+V2+V3+V5 | 7 | 0.9161 | 0.6793 | 0.6698 |

Table S1c. Top-3 lead configurations per input count N ranked by Composite Lead Score (CLS, α = 0.50). CLS = 0.50 × (F1 / 0.6751) + 0.50 × (1 − Eₙₒᵣᴹ), where Eₙₒᵣᴹ = (E − 1) / 9. Higher CLS reflects a better accuracy–efficiency balance. ★ marks the Kneedle-detected efficiency–accuracy knee (N = 4).

| **N** | **Rank** | **Configuration** | **E** | **Mean PCC** | **Macro-F1** | **CLS (α = 0.50)** |
| --- | --- | --- | --- | --- | --- | --- |
| 1 | 1 | **V6** | 1 | 0.7101 | 0.5211 | **0.8859** |
|  | 2 | V5 | 1 | 0.7119 | 0.5190 | 0.8844 |
|  | 3 | V4 | 1 | 0.7025 | 0.4707 | 0.8486 |
| 2 | 1 | **V1+V6** | 2 | 0.7373 | 0.5819 | **0.8754** |
|  | 2 | V2+V6 | 2 | 0.7526 | 0.5769 | 0.8717 |
|  | 3 | V2+V5 | 2 | 0.7531 | 0.5765 | 0.8714 |
| 3 | 1 | **V1+V2+V6** | 3 | 0.7626 | 0.5975 | **0.8314** |
|  | 2 | V1+V3+V6 | 3 | 0.7577 | 0.5950 | 0.8296 |
|  | 3 | V1+V2+V5 | 3 | 0.7635 | 0.5943 | 0.8291 |
| ★4 | 1 | **I+III+AVR+AVL** | 4 | 0.8515 | 0.6340 | **0.8029** |
|  | 2 | I+II+III+AVR | 4 | 0.8510 | 0.6337 | 0.8027 |
|  | 3 | I+II+AVR+AVL | 4 | 0.8508 | 0.6324 | 0.8017 |
| 5 | 1 | **I+II+AVR+AVL+AVF** | 4 | 0.8325 | 0.6378 | **0.8057** |
|  | 2 | II+III+AVR+AVL+AVF | 4 | 0.8312 | 0.6321 | 0.8015 |
|  | 3 | I+II+III+AVL+AVF | 4 | 0.8316 | 0.6312 | 0.8008 |
| 6 | 1 | **I+II+III+AVR+AVL+AVF** | 4 | 0.8070 | 0.6339 | **0.8028** |
|  | 2 | I+III+AVR+AVL+AVF+V2 | 5 | 0.8812 | 0.6608 | 0.7672 |
|  | 3 | I+II+AVR+AVL+AVF+V2 | 5 | 0.8801 | 0.6597 | 0.7664 |
| 7 | 1 | **I+II+III+AVR+AVL+AVF+V1** | 5 | 0.8411 | 0.6606 | **0.7670** |
|  | 2 | I+II+III+AVR+AVL+AVF+V2 | 5 | 0.8601 | 0.6604 | 0.7669 |
|  | 3 | I+II+III+AVR+AVL+AVF+V3 | 5 | 0.8676 | 0.6545 | 0.7625 |
| 8 | 1 | **I+II+III+AVR+AVL+AVF+V1+V6** | 6 | 0.8631 | 0.6727 | **0.7205** |
|  | 2 | I+II+III+AVR+AVL+AVF+V2+V5 | 6 | 0.8848 | 0.6711 | 0.7192 |
|  | 3 | I+II+III+AVR+AVL+AVF+V2+V4 | 6 | 0.8831 | 0.6707 | 0.7190 |

**Table S2. Disease-specific optimal lead configurations per input count N. For each of the five PTB-XL diagnostic superclasses (NORM, MI, STTC, CD, HYP) and each N = 1–11, the configuration achieving the highest per-class F1 is listed. E = electrode contact count. All values are the best achievable across LR, Ridge, and LightCNN. For N ≥ 9 the configuration is written as All−{excluded leads} for brevity. ★ marks the Kneedle-detected efficiency–accuracy knee (N = 4).**

| **Disease** | **N** | **Best Configuration** | **E** | **Best F1** |
| --- | --- | --- | --- | --- |
| NORM | 1 | AVR | 4 | 0.7946 |
|  | 2 | I+AVF | 4 | 0.8349 |
|  | 3 | II+AVR+V6 | 5 | 0.8397 |
|  | ★4 | I+AVL+AVF+V2 | 5 | 0.8482 |
|  | 5 | II+AVF+V1+V2+V3 | 7 | 0.8481 |
|  | 6 | I+AVR+AVL+V2+V4+V6 | 7 | 0.8505 |
|  | 7 | I+III+AVR+V1+V2+V3+V6 | 8 | 0.8522 |
|  | 8 | I+III+AVR+V1+V2+V3+V4+V6 | 9 | 0.8525 |
| MI | 1 | **III** | 4 | 0.4923 |
|  | 2 | III+V3 | 5 | 0.6087 |
|  | 3 | I+AVF+V2 | 5 | 0.6736 |
|  | ★4 | I+AVL+AVF+V2 | 5 | 0.6794 |
|  | 5 | I+AVL+AVF+V2+V5 | 6 | 0.6808 |
|  | 6 | I+III+AVL+AVF+V2+V3 | 6 | 0.6815 |
|  | 7 | I+III+AVR+AVF+V2+V3+V5 | 7 | 0.6799 |
|  | 8 | I+II+III+AVR+AVF+V2+V3+V5 | 7 | 0.6761 |
| STTC | 1 | **V5** | 1 | 0.6856 |
|  | 2 | V3+V5 | 2 | 0.7205 |
|  | 3 | I+V2+V5 | 6 | 0.7413 |
|  | ★4 | AVL+V3+V5+V6 | 7 | 0.7527 |
|  | 5 | I+II+V4+V5+V6 | 7 | 0.7543 |
|  | 6 | II+AVL+V2+V4+V5+V6 | 8 | 0.7561 |
|  | 7 | II+AVR+AVL+V3+V4+V5+V6 | 8 | 0.7559 |
|  | 8 | II+AVR+AVL+V2+V3+V4+V5+V6 | 9 | 0.7599 |
| CD | 1 | **II** | 4 | 0.6267 |
|  | 2 | II+V1 | 5 | 0.7003 |
|  | 3 | II+V1+V2 | 6 | 0.7138 |
|  | ★4 | II+AVL+AVF+V1 | 5 | 0.7189 |
|  | 5 | II+III+V1+V4+V5 | 7 | 0.7215 |
|  | 6 | II+III+AVF+V1+V4+V5 | 7 | 0.7211 |
|  | 7 | II+III+AVR+AVL+V1+V4+V6 | 7 | 0.7189 |
|  | 8 | II+III+AVR+AVL+AVF+V1+V4+V6 | 7 | 0.7180 |
| HYP | 1 | **V6** | 1 | 0.4720 |
|  | 2 | I+V6 | 5 | 0.4676 |
|  | 3 | I+V1+V6 | 6 | 0.4735 |
|  | ★4 | I+V2+V5+V6 | 7 | 0.4791 |
|  | 5 | II+AVF+V2+V3+V5 | 7 | 0.4795 |
|  | 6 | I+III+V1+V2+V3+V6 | 8 | 0.4739 |
|  | 7 | I+II+V1+V2+V3+V5+V6 | 9 | 0.4701 |
|  | 8 | I+II+III+V1+V2+V3+V4+V5 | 9 | 0.4696 |
